# A revision of the continental species of *Copa* Simon, 1885 (Araneae, Corinnidae) in the Afrotropical Region

**DOI:** 10.3897/zookeys.276.4233

**Published:** 2013-03-06

**Authors:** Charles Richard Haddad

**Affiliations:** 1 Department of Zoology & Entomology, University of the Free State, P.O. Box 339, Bloemfontein 9300, South Africa

**Keywords:** Castianeirinae, cryptic, ground-dwelling, litter, lycosiform, *nomen dubium*, spider, taxonomy

## Abstract

The cryptic ground-dwelling castianeirine genus *Copa* Simon, 1885 (Araneae: Corinnidae) is revised in the continental Afrotropical Region. The type species of the genus, *Copa flavoplumosa* Simon, 1885, is redescribed and considered a senior synonym of *Copa benina* Strand, 1916 **syn. n.** and *Copa benina nigra* Lessert, 1933 **syn. n.** It is widespread throughout the Afrotropical Region but has not been introduced to any of the associated regional islands. A new species, *Copa kei* **sp. n.**, is described from South Africa. *Copa agelenina* Simon, 1910, originally described from a subadult female from southern Botswana, is considered a *nomen dubium*. *Copa flavoplumosa* is a characteristic species of leaf litter spider assemblages and is particularly prevalent in savanna habitats on the continent, but also occurs in various forest types, grasslands, fynbos and semi-arid Nama Karoo habitats. In contrast, *Copa kei* **sp. n.** has only been recorded from Afromontane and coastal forests in south-eastern South Africa.

## Introduction

The spider genus *Copa* Simon, 1885 (Araneae: Corinnidae) is only known from the Afrotropical and Palaearctic Regions, and some of the species from the latter have recently been studied by Deeleman-Reinhold (1995, 2001). Deeleman-Reinhold (2001) described the genus *Echinax* to include three species of *Copa* from South-East Asia that she had earlier described in 1995, and an additional new species. Yang et al. (2004) subsequently described a fifth species of *Echinax* from China. Thus, only two Asian species of *Copa* remain, both described from Sri Lanka (Simon 1896). Prior to this revision, seven species and one subspecies of *Copa* were known from the Afrotropical Region (Dippenaar-Schoeman and Jocqué 1997), of which one described by Simon (1909) has been transferred to *Echinax* (Haddad 2012a). Another species, described by Strand (1916), will be transferred to a new castianeirine genus (Haddad 2012b in prep.).

**Figures 1–6.**
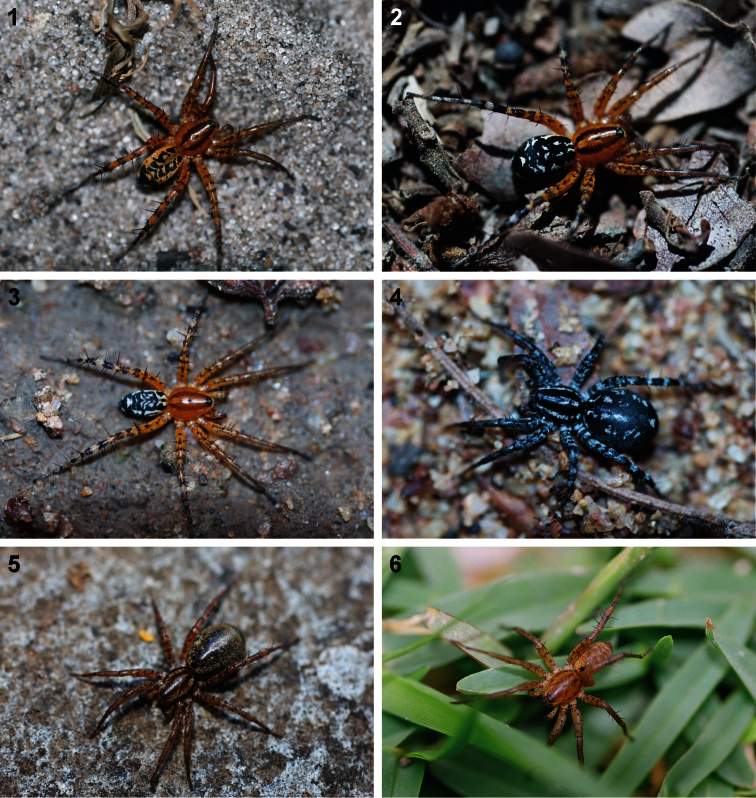
General habitus photographs of *Copa flavoplumosa* Simon, 1885 **(1–4)** and *Copa kei* sp. n. **(5, 6)**: **1** female from Lesideng Research Camp, Botswana **2** female from Livingtone, Zambia **3** male and **4** female from Wildlives Game Farm, Zambia **5** female from Hogsback, South Africa **6** male from Cwebe Nature Reserve, South Africa.

Although most genera in this subfamily resemble ants, *Copa* species have cryptic colouration and closely resemble wolf spiders of the family Lycosidae (Figs 1–6), a characteristic shared with *Echinax* and two undescribed lycosiform castianeirine genera (Haddad 2012a, b, in prep.). *Copa* are very common spiders in the leaf litter of various habitats and are predominantly ground-living, occurring widely in savanna woodlands but also occasionally in forests, where they are well camouflaged. They usually share litter microhabitats with species of several other castianeirine genera, including *Cambalida* Simon, 1909, *Merenius* Simon, 1909 and *Castianeira* Keyserling, 1879 (Haddad 2012b, 2012c). *Copa flavoplumosa* Simon, 1885 is also recorded here from drier habitats, including fynbos, grassland and Nama Karoo in South Africa and the arid savannas of north-western South Africa, Botswana and Angola, thereby showing considerable ecological flexibility and adaptability. In contrast, the species of the other three cryptic lycosiform genera are primarily arboreal and are rarely collected in leaf litter.

The current study presents the first revision of the continental Afrotropical species of the genus, and the Madagascan fauna, including two species described by Simon (1903) and Strand (1907) and nearly 30 new species, will be treated at a later stage in a separate paper.

## Material and methods

All specimens examined during this study were observed in 70% ethanol using a Nikon SMZ800 stereomicroscope for descriptions, digital photographs and measurements. A series of digital photographs of the male emboli of eachspecies were taken using a Nikon Coolpix 8400 mounted on a Nikon SMZ800 stereomicroscope. The photographs were then stacked using Combine ZM software (http://www.hadleyweb.pwp.blueyonder.co.uk ) to increase depth of field. Photographs of live *Copa flavoplumosa* and *Copa kei* sp. n. were taken in the field using a Canon EOS 40D digital camera with 50mm or 100mm macro lenses. Material of both aforementioned species was prepared for scanning electron microscopy by dehydrating the specimens in a graded ethanol series and critical-point drying them in an argon chamber, after which they were fixed to aluminium stubs and sputter-coated with gold three times for three minutes. The material was then studied in a JEOL 6400 WinSEM and digital micrographs were taken.

All measurements are given in millimetres (mm). Total body length measurements are given for the smallest and largest specimens of each sex to give an indication of size variation, and body, eye and leg measurements are given for the specimens indicated in the descriptions. Descriptions of the eye arrangements are given for the anterior view of the anterior eye row and dorsal view of the posterior eye row. The epigynes and male palps of each species were dissected, cleaned in a Labcon 5019U ultrasonic bath in 70% ethanol for 30 seconds, and drawn. Scale bars were added to all microscope photographs and illustrations in Corel Draw 14.0.

Abbreviations used in the descriptions are as follows: AER – anterior eye row; AL – abdomen length; ALE – anterior lateral eye; ALS – anterior lateral spinneret(s); AME – anterior median eye; AW – abdomen width; CL – carapace length; CW – carapace width; FL – fovea length; MOQ – median ocular quadrangle; MOQAW – median ocular quadrangle anterior width; MOQL – median ocular quadrangle length; MOQPW – median ocular quadrangle posterior width; PER – posterior eye row; PERW – posterior eye row width; PLE – posterior lateral eye; PLS – posterior lateral spinneret(s); PME – posterior median eye; PMS – posterior median spinneret(s); SL – sternum length; ST – spermatheca; SW – sternum width; TL – total length. Leg spination follows the format of Bosselaers and Jocqué (2000) and includes the following abbreviations: do – dorsal; pl – prolateral; plv – prolateral ventral; rl – retrolateral; rlv – retrolateral ventral; vt – ventral terminal.

The material examined in this study is deposited in the following institutions (curators given in parenthesis):

**AMNH **American Museum of Natural History, New York, USA (Norman Platnick)

**BMNH** British Museum of Natural History, London, UK (Janet Beccaloni)

**CAS** California Academy of Sciences, San Francisco, USA (Charles Griswold)

**MNHG** Museum of Natural History, Geneva, Switzerland (Peter Schwendinger)

**MNHN** Museum National d’Histoire Naturelle, Paris, France (Christine Rollard)

**MRAC** Royal Museum for Central Africa, Tervuren, Belgium (Rudy Jocqué)

**NCA** National Collection of Arachnida, ARC – Plant Protection Research Institute, Pretoria, South Africa (Ansie Dippenaar-Schoeman)

**NMBA** National Museum, Bloemfontein, South Africa (Leon Lotz)

**NMSA** KwaZulu-Natal Museum, Pietermaritzburg, South Africa (Audrey Ndaba)

**NMZA** National Museum of Zimbabwe, Bulawayo, Zimbabwe (Moira FitzPatrick)

**PCRS** Personal collection of Tony Russell-Smith, Sittingbourne, UK

**SAMC** Iziko South African Museum, Cape Town, South Africa (Margie Cochrane)

**TMSA** Ditsong National Museum of Natural History, Pretoria, South Africa (Robin Lyle)

**ZMB** Zoologisches Museum, Berlin, Germany (Jason Dunlop)

**ZMUC **Zoological Museum, University of Copenhagen, Denmark (Nikolaj Scharff)

Where locality co-ordinates were not provided on specimen labels or were not available in the institutional databases, they were traced using the Global Gazetteer Version 2.2 (www.fallingrain.com ) and are indicated in square brackets. Distribution maps were produced using the online mapping software SimpleMappr (Shorthouse 2010).

## Taxonomy

### Family Corinnidae Karsch, 1880. Subfamily Castianeirinae Reiskind, 1969

#### Genus Copa

Simon, 1885

http://species-id.net/wiki/Copa

Copa Simon 1885: 395; Simon 1897: 173; Reiskind 1969: 165; Dippenaar-Schoeman and Jocqué 1997: 128; Deeleman-Reinhold 2001: 359.

##### Type species:

*Copa flavoplumosa* Simon, 1885, by monotypy.

##### Diagnosis.

*Copa* can be recognised from other cryptic lycosiform Castianeirinae, particularly *Echinax* and two undescribed Afrotropical lycosiform genera, by the presence of fine proximal and distal dorsal setae on the anterior patellae (Figs 24, 34), and proximal and distal spines on the posterior patellae (Fig. 35), which are clearly shorter than the particular leg segment; in *Echinax* all of the patellae have well-developed spines that are longer than the patella, and in the two undescribed genera the proximal structure on the posterior patellae is a fine seta rather than a spine, as is the case in *Copa*. Further, the AME are approximately 1.25–1.50 times ALE diameter in *Copa* (Figs 14, 15, 19), while at least twice the diameter in *Echinax* and 1.25 times or less in the two undescribed genera. Lastly, the carapace of *Copa* is 3.30–3.75 times broader than the PER, while less than 3 times broader in the other three genera. These characteristics are also applicable to immature specimens.

##### Description.

**Figures 7–12.**
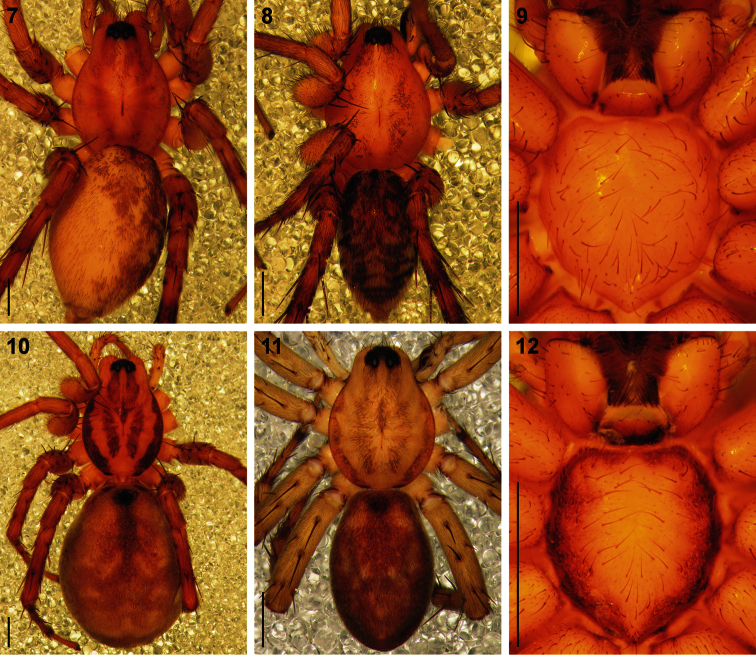
Digital microscope photographs of *Copa flavoplumosa* Simon, 1885 from D.R. Congo **(7–9)** and *Copa kei* sp. n.from South Africa **(10–12)**: **7, 10** female, dorsal habitus **8, 11** male, dorsal habitus **9, 12** sternum of female in ventral view. Scale bars = 1.0 mm.

**Figures 13–24.**
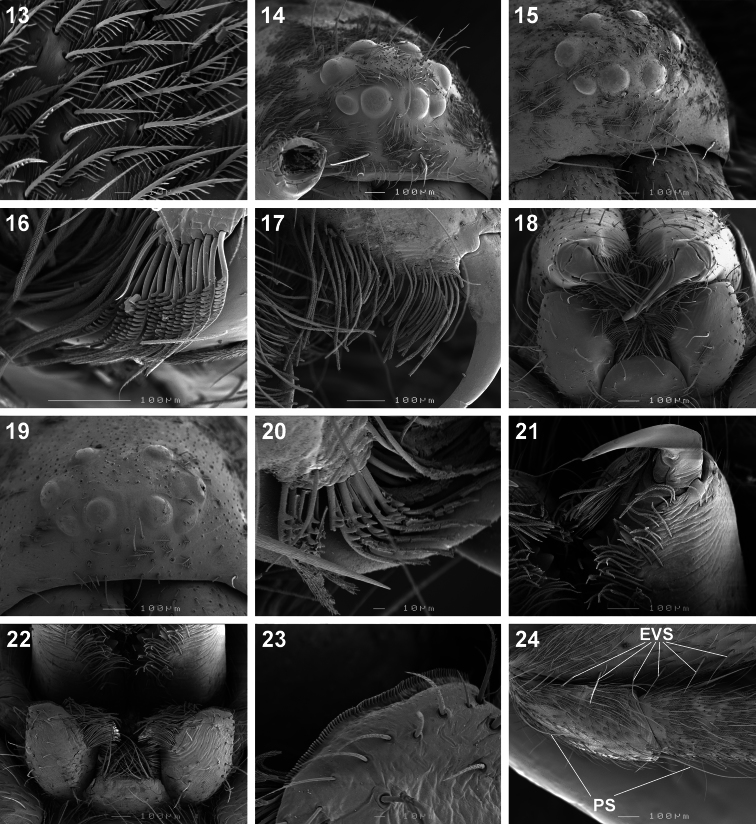
Scanning electron microscope photographs of *Copa flavoplumosa* Simon, 1885 female **(13, 14, 16)** and male **(15, 17, 18)** and *Copa kei* sp. n. female **(19–24)**: **13** dorsal carapace setae **14, 15, 19** eye region and clypeus, anterolateral **(14, 15)** and anterior **(19)** views **16, 17, 20** cheliceral promarginal bent setae, anterior view **18, 22** mouthparts, ventral view **21** chelicerae, ventral view **23** serrula **24** femur, patella and tibia of leg II, indicating erect ventral setae on femora **(EVS)** and proximal and distal dorsal patellar setae **(PS)**.

**Figures 25–30.**
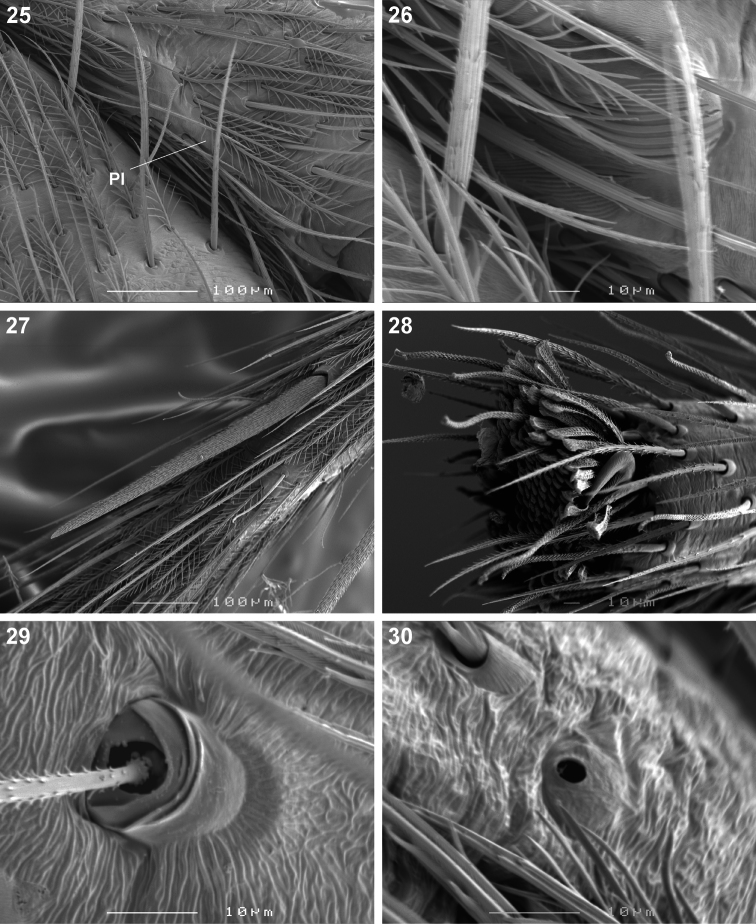
Scanning electron microscope photographs of *Copa kei* sp. n. female: **25** patella II, indicating patellar indentation **(PI)** **26** same, detail of lyriform organ at proximal end of PI **27** metatarsus IV, spine and setae **28** tarsus III, tarsal claw and claw tuft **29** tarsus IV, trichobothrium base **30** same, tarsal organ.

**Figures 31–36.**
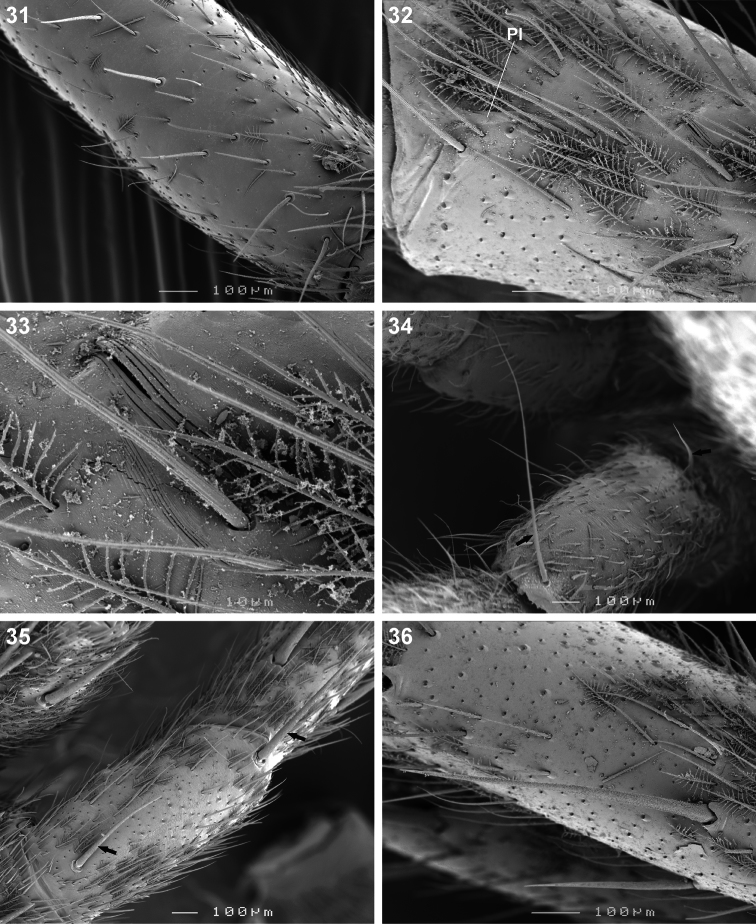
Scanning electron microscope photographs of *Copa flavoplumosa* Simon, 1885 male **(31)** and female **(32–36)**: **31** femur I, erect ventral setae **32** patella II, indicating patellar indentation **(PI)** **33** same, detail of lyriform organ at proximal end of PI **34** patella II, arrows indicating proximal and distal dorsal patellar setae **35** patella III, arrows indicating proximal and distal dorsal patellar spines **36** tibia II, spines and feathery setae.

**Figures 37–42. F6:**
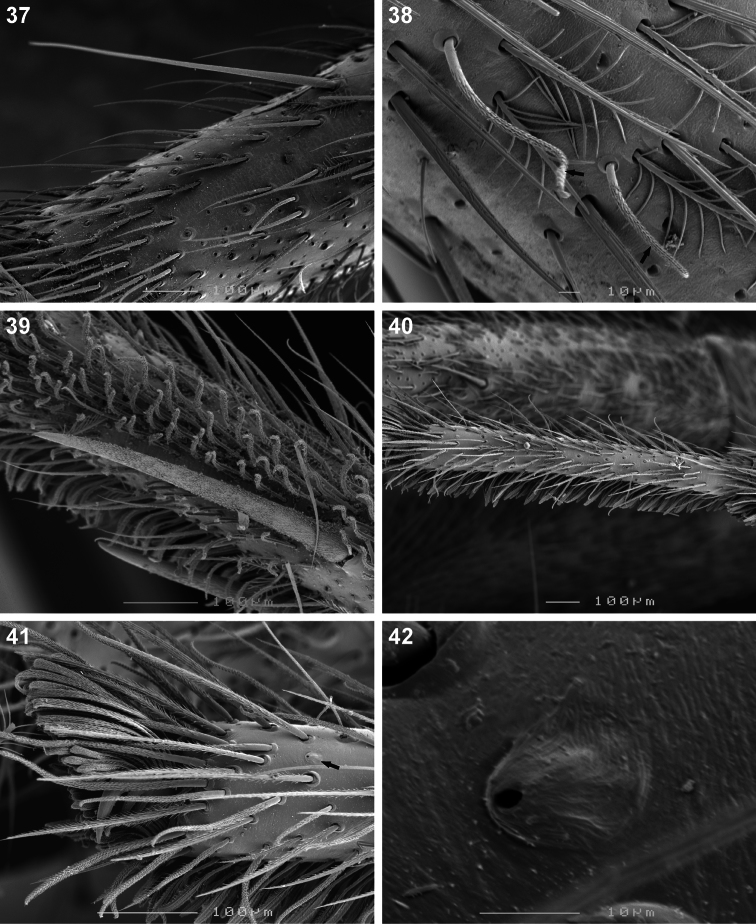
Scanning electron microscope photographs of *Copa flavoplumosa* Simon, 1885 female **(37, 39–42)** and male **(38)**: **37** tibia I, long dorsal seta **38** tibia I, arrows indicating short erect setae **39** metatarsus II, spines and scopula **40** tarsus III **41** same, claw tuft and tarsal organ (arrow) **42** same, tarsal organ.

**Figures 43–48.**
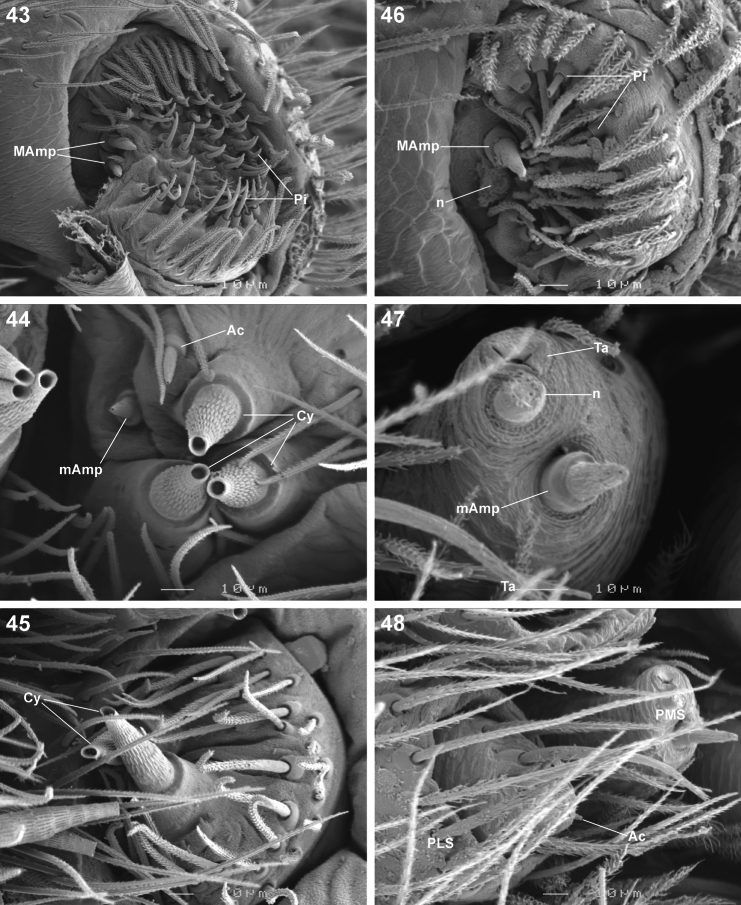
Scanning electron microscope photographs of *Copa flavoplumosa* Simon, 1885 female **(43–45)** and male **(46–40)** spinneret morphology: **43, 46** anterior lateral spinneret **44, 47** posterior median spinneret **45, 48** posterior lateral spinneret. Abbreviations: **Ac** aciniform gland spigot(s) **Cy** cylindrical gland spigot(s) **MAmp** major ampullate gland spigot(s) **mAmp** minor ampullate gland spigot(s) **n** nubbin **Pi** piriform gland spigot(s) **PLS** posterior lateral spinneret **PMS** posterior median spinneret **ta** tartipore.

**Figures 49–54.**
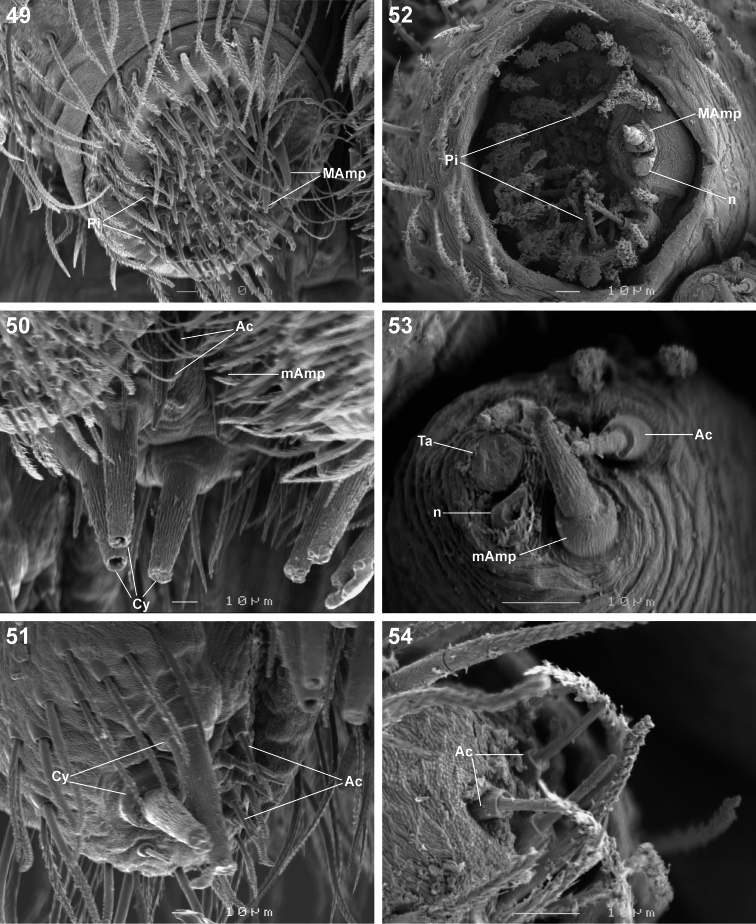
Scanning electron microscope photographs of *Copa kei* sp. n. female **(49–51)** and male **(52–54)** spinneret morphology: **49, 52** anterior lateral spinneret **50, 53** posterior median spinneret **51, 54** posterior lateral spinneret. Abbreviations: **Ac** aciniform gland spigot(s) **Cy** cylindrical gland spigot(s) **MAmp** major ampullate gland spigot(s) **mAmp** minor ampullate gland spigot(s) **n** nubbin **Pi** piriform gland spigot(s) **ta** tartipore.

**Figures 55–60.**
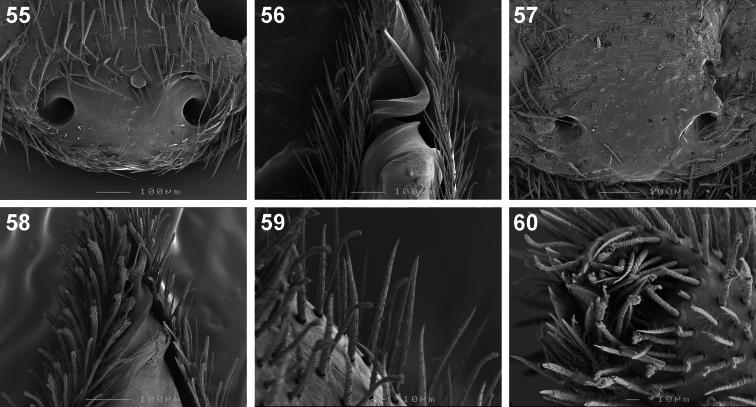
Scanning electron microscope photographs of *Copa flavoplumosa* Simon, 1885 **(55, 56)** and *Copa kei* sp. n. **(57–60)**: **55, 57** female epigyne, ventral view **56, 58** male embolus, ventral view **59** male palpal cymbial setae **60** distal end of cymbium, retrolateral distal view.

**Figures 61–66.**
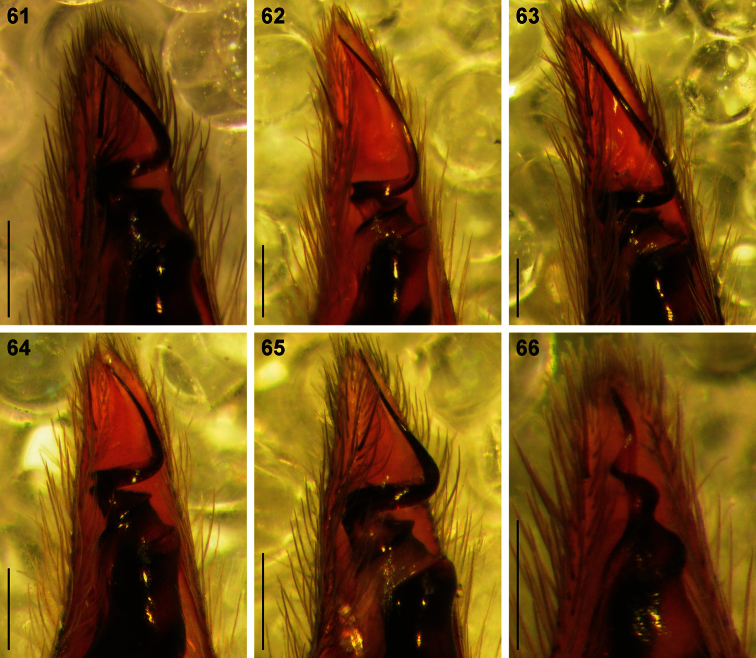
Digital microscope photographs of emboli of *Copa* species in ventral view: **61–65** *Copa flavoplumosa* Simon, 1885 from D.R. Congo **(61)**, Cameroon **(62)**, Tanzania **(63)**, Botswana **(64)** and South Africa **(65)** **66** *Copa kei* sp. n. from South Africa. Scale bars = 0.1 mm.

Medium-sized spiders, 5.20–9.80mm in length; carapace usually pale yellow to dark orange-brown with black markings, rarely black with white markings (Figs 1–8, 10, 11); carapace surface smooth, with black feathery setae covering markings (Fig. 13); several long curved setae on clypeus, eye region and posterior to PER up to midpoint (Figs 14, 15, 19); carapace oval, broadest at posterior of coxae II, eye region narrow, fovea distinct; posterior margin very slightly concave or straight (Figs 7, 8, 10, 11). AER procurved, AME approximately 1¼ to 1½ times ALE diameter; AME separated by ½ or less their diameter, nearly touching ALE (Figs 14, 15, 19); PER strongly procurved, PME very slightly larger than PLE; PME closer to PLE than to each other; MOQ width equal anteriorly and posteriorly, or very slightly broader posteriorly, longer than wide. Chilum distinct, triangular, bilateral with clear median separation; cheliceral promargin with two or three teeth, retromargin with two teeth; shaggy seta absent; curved setae on cheliceral promargin pectinate in females (Figs 16, 20) and finely plumose in males (Fig. 17); endites straight laterally, with distinct serrula comprising sharp, ventrally curved denticles, with dense maxillar hair tuft on mesal margins (Figs 18, 22, 23); labium hemispherical, wider than long. Pleural bars sclerotised, isolated; sternum slightly longer than broad, shield-shaped, slightly narrowed anteriorly, with or without markings; surface smooth, densely covered in feathery setae, with many long erect straight setae; precoxal triangles present, intercoxal sclerites only present between coxae I and II (Figs 9, 12). Leg formula 4132 in females, 4312 or 4123 in males, legs I and III nearly equal in length; legs strongly spined, femora, patellae, tibiae and metatarsi covered in short straight black setae and black and white feathery setae (Figs 24–27, 31–39), feathery setae sparse on tarsi; retrocoxal window absent; femora with several scattered erect ventral setae (Figs 24, 31); anterior patellae with proximal and distal long fine dorsal setae (Figs 24, 34); posterior patellae with fine long proximal dorsal seta and distal spine, clearly shorter than patella (Fig. 35); patellar indentation narrow, broad at proximal end (Figs 25, 26, 32, 33); metatarsi III longer than metatarsi I and II; metatarsi distally scopulate (Fig. 39); tibiae, metatarsi and tarsi with several dorsal and lateral trichobothria with sunken distal plate (Fig. 29), also with several short erect setae (Fig. 38); tarsal organ oval, slightly elevated from integument, surface finely wrinkled, opening oval (Figs 30, 42); paired tarsal claws short, with very dense claw tufts in between (Figs 28, 41); metatarsi III and IV without terminal preening brush or comb. Abdomen oval, either yellow-orange with black markings or black with white markings; three pairs of fine straight setae on anterior margin above pedicel; dorsal scutum small, strongly sclerotized, extending less than ⅛ abdomen length in females and slightly more than ½ abdomen length in males; two pairs of distinct sclerotised dorsal sigilla present in both sexes (Figs 7, 8, 11, 12); epigastric region moderately sclerotised, venter without post-epigastric sclerites and ventral sclerite, inframamillary sclerite present, distinct, densely covered in short setae; two paired rows of tiny sclerites from epigastric furrow to spinnerets, outer row weakly sclerotised and indistinct. Spinnerets: ALS of female (Figs 43, 49) with two major ampullate gland spigots and many piriform gland spigots; ALS of male (Figs 44, 50) with single major ampullate gland spigot, single large adjacent nubbin and many piriform gland spigots; PMS of female (Figs 45, 51) with three large cylindrical gland spigots, one small minor ampullate gland spigot and one or two aciniform gland spigots; PMS of male (Figs 46, 52) with one large minor ampullate gland spigot, one tartipore and one nubbin, with zero aciniform gland spigots in *Copa flavoplumosa* and two in *Copa kei* sp. n.; PLS of female (Figs 47, 53) with two large cylindrical gland spigots and zero (in *Copa flavoplumosa*) or several (in *Copa kei* sp. n.) aciniform gland spigots; PLS of *Copa flavoplumosa* male with two reduced aciniform gland spigots and several tiny nubbins present (Fig. 48); PLS of *Copa kei* sp. n. with three aciniform gland spigots only (Fig. 54). Female epigyne with 6-shaped or curved sclerotized epigynal ridges leading to lateral copulatory openings (Figs 55, 57); copulatory ducts directed anteriorly or transversely before entering ST II posteriorly; ST II usually oval, sometimes expanded posterolaterally, connected broadly to somewhat elongated posterior ST I. Male palpal segments without apophyses; cymbium with spines prolaterally and ventrally, dorsal surface covered in curved finely plumose setae with round tip and thicker straight setae with sharp tips (Fig. 59); distal end of cymbium in *Copa kei* sp. n. with shallow depression, densely covered in setae (Fig. 60); embolus with variable width and angle of base, and length and curvature of distal coil (Figs 56, 58, 61–66).

##### Key to the continental Afrotropical species of *Copa*

**Table:** 

1	Males	2
–	Females	3
2	Embolus with broad base and long curved distal section (Fig. 56)	*Copa flavoplumosa* Simon, 1885
–	Embolus with narrow base and narrow coiled distal section (Fig. 58)	*Copa kei* sp. n.
3	Epigyne with large 6-shaped epigynal ridges with lateral copulatory openings (Fig. 55); entrance ducts directed anteriorly, with distinct loop before entering ST II (Fig. 68)	*Copa flavoplumosa* Simon, 1885
–	Epigyne with small cup-shaped epigynal ridges covering copulatory openings (Fig. 57); entrance ducts directed transversely and slightly anteriorly before entering ST II (Fig. 74)	*Copa kei* sp. n.

#### Copa flavoplumosa

Simon, 1885

http://species-id.net/wiki/Copa_flavoplumosa

Figures 1–4 7–9, 12 –18 31 [Fig F6] –48 55, 56 61–65 67–70 71

Copa flavoplumosa Simon 1885: 396; Simon 1897: 168, 173, fig. 159.
Copa flavopilosa Simon 1897: 160, fig. 159 (misspelling).
Copa benina Strand 1916: 93; Lessert 1921: 429, figs 66–69 syn. n.
Copa benina nigra Lessert 1933: 129, fig. 48 syn. n.

##### Type material.

Female lectotype and one female paralectotype, here designated, together with one non-type male: ANGOLA: Landana [05°13'S, 12°08'E], MNHN 5338 (examined).

**Type material of synonyms.** *Copa benina* Strand, 1916. Female holotype. D.R. CONGO: Fort Beni [00°29'N, 29°27'E], Ruwenzori, leg. Expedition Adolf Friedrich Herzog von Mecklenburg, I.1908, ZMB 28199 (examined); *Copa benina nigra* Lessert, 1933. Syntypes? ANGOLA: one male from Chimporo and one female from Rio Mbale, MNHG (examined).

##### Other material examined.

BOTSWANA: *Okavango Delta*: Airstrip near Delta Camp, 19°32'S, 23°05'E, leg. K. Wilkins, 13.I.2001 (bush beating), 1♂ (NMZA 14085); Lesideng Research Camp, Near Shakawe, 18°25.822'S, 21°53.771'E, leg. C. Haddad, 25.XI.2006 (leaf litter), 1♀ (NCA 2007/936); Same locality, leg. C. Haddad, 26–29.XI.2006 (under bark), 1♀ (NCA 2007/987); Maun [19°59'S, 23°25'E], leg. A. Russell-Smith, 27.X.1978 (in deep litter, riverine forest), 2♂ (BMNH); Same locality, Government Camp house 36 [19°59'S, 23°25'E], leg. A. Russell-Smith, I–II.1977, 1♀ (BMNH); Botswana, Maun, Maphaneng Pan [19°55'S, 23°26'E], leg. A. Russell-Smith, 8.II.1976 (riverine woodland, leaf litter), 2♂ (BMNH); Moremi Game Reserve [19°15'S, 23°05'E], leg. W. & I. Barnard, 13–24.I.1991 (mopane woodland), 1♀ (NCA 91/985); Moremi Game Reserve, Maxwee [19°28'S, 23°39'E], leg. A. Russell-Smith, 2.I.1976 (mopane woodland), 1♂ 1♀ (PCRS); Samochima lagoon, Shakawe Fishing Camp, 18°25.749'S, 21°54.035'E, leg. C. Haddad, 10.XII.2006 (leaf litter), 1♂ (NCA 2007/1051); “Woody” Island, NW of Xugana Island, 19°04'S, 23°03'E, leg. B.H. Lamoral, 21–22.XI.1980, 2♂ (NMSA); Xugana Island, 130km NNW of Maun, 19°04'S, 23°03'E, leg. B.H. Lamoral, 18–21.XI.1980, 1♂ (NMSA); Same data (forest floor and logs), 4♂ 2♀ (NMSA); Same locality, leg. B.H. Lamoral, 22–24.XI.1980, 1♀ (NMSA); Same data, 1♀ (NMSA). *North-East Region*: Near Francistown, Selkirk Mine, 21°19.332'S, 27°44.148'E, leg. D.H. Jacobs, 28.III–5.IV.2008, 1♀ (NCA 2008/2905). CAMEROON: Bali, Bafuchu Mbu, Shum Laka, 05°51'N, 10°05'E, 1600m a.s.l., leg. H. Doutrelepont, XII.1991–II.1992 (pitfall), 1♂ (MRAC 174794); Chabal Mbabo, South-western slope, 07°25'N, 12°49'E, 1250m a.s.l., leg. Bosmans & Van Stalle, 7–13.IV.1983 (grassland with shrubs, pitfalls), 1♂ (MRAC 162220); Same locality, 1500m a.s.l., leg. Bosmans & Van Stalle, 11.IV.1983 (gallery forest, litter), 1♀ (MRAC 162222); Ebolowa, Nkoumvom [02°55'N, 11°09'E], leg. M.C. Day, 1980 (pitfall traps), 1♂ (BMNH); Faro Game Reserve, 08°24'N, 12°49'W, leg. R. Jocqué, K. Loosveldt, L. Baert & M. Alderweireldt, 3.V.2007 (river bed, pitfall), 1♀ (MRAC 221128); Same locality, leg. R. Jocqué, K. Loosveldt, L. Baert & M. Alderweireldt, 2.V.2007 (gallery forest, pitfall), 2♂ (MRAC 221134); Same data, 4.V.2007, 1♂ 2♀ (MRAC 221169); Same data, 5.V.2007, 3♂ 1♀ (MRAC 221229); Same locality, leg. R. Jocqué, K. Loosveldt, L. Baert & M. Alderweireldt, 27.IV.2007 (gallery forest, sieving), 1♀ (MRAC 221434); Same locality, leg. R. Jocqué, K. Loosveldt, L. Baert & M. Alderweireldt, 1.V.2007 (mature gallery forest, by hand), 1♀ (MRAC 221359); Same locality, leg. R. Jocqué, K. Loosveldt, L. Baert & M. Alderweireldt, 5.V.2007 (mature gallery forest, pitfall), 6♂ 2♀ (MRAC 221211); Same locality, leg. R. Jocqué, K. Loosveldt, L. Baert & M. Alderweireldt, 26.IV.2007 (litter, by hand), 3♀ (MRAC 221280); Same locality, leg. R. Jocqué, K. Loosveldt, L. Baert & M. Alderweireldt, 19.IV.2007 (wooded savanna, beating), 1♂ (MRAC 221407); Same locality, leg. R. Jocqué, K. Loosveldt, L. Baert & M. Alderweireldt, 3.V.2007 (litter under tree, by hand), 1♂ 1♀ (MRAC 221432); Mbam mountain area, near Katoupi, Western slope, 05°54'N, 10°44'E, 1550m a.s.l., leg. Bosmans & Van Stalle, 31.III.1983 (gallery forest), 2♀ (MRAC 162244). CENTRAL AFRICAN REPUBLIC: Bambari, 04°15'N, 21°54'E, leg. G. Pierrard, II.1969, 1♂ (MRAC 136635). D.R. CONGO: Mikembo, 11°28'S, 27°39'E, leg. M. Hasson, 26.XI.2010 (miombo woodland, Uapaca forest, pitfall traps), 1♂ (MRAC 234447), 1♂ 1♀ (MRAC 234384); Same locality, leg. M. Hasson, 26.XI.2010 (gallery forest, alongside river, pitfall traps), 7♂ 4♀ (MRAC 234461); Parc National Albert, Northern Sector, Talya River, area to the right of Lume, near Mutsora [00°19'N, 29°45'E], 1140m a.s.l., leg. P. van Schuytbroeck, 14.II.1955, 1♀ (MRAC 234182); Tshopo, Masako Forest, 15 km N of Kisangani, 00°35'N, 25°11'E, leg. L. de Vos, 19–27.I.1998, 1♂ (MRAC 169357); Same locality, leg. J.-L. Juakaly, 17.XII.2002 (pitfalls, young fallow), 1♀ (MRAC 214341); Same data, 2♂ (MRAC 214334); Same locality, leg. J.-L. Juakaly, 2.VII.2002 (young fallow, pitfall), 1♂ (MRAC 214363). ETHIOPIA: Yayu Coffee Forest, 08°23'N, 35°48'E, leg. N. Aklilu, 15.II.2004 (secondary forest, look down), 1♂ 1♀ (MRAC 229596); Same locality, leg. N. Aklilu, 14.XI–12.XII.2003 (pitfall trap), 1imm. 2♂ 4♀ (MRAC 220773); Same locality, leg. N. Aklilu, 2004 (sieving, plantation), 1♂ (MRAC 230893). GABON: Estuaire, Ntoum, 00°23'N, 09°47'E, leg. A. Pauly, 5–15.X.1985 (pelouse jardin, pièges moericke), 1♂ (MRAC 172826); Same locality, leg. A. Pauly, X–XI.1985 (piège bac d'Eau, forêt), 1♂ (MRAC 172837); Same locality, leg. A. Pauly, 7.XI.1985 (carrier de sable, piège bac d'Eau), 1♂ (MRAC 172934). GUINEÉ: F.C. de Ziama, 08°24'N, 09°17'W, leg. D. Flomo, 26.XII.1998 (pitfalls, rain forest), 1♂ (MRAC 216225); Same data, 8.I.1999, 1♂ (MRAC 216208); Same data, 21.I.1999, 2♀ (MRAC 216209); Same data, 15.II.1999, 1♀ (MRAC 216226). IVORY COAST: Appouesso, F.C. Bossematié, 06°35'N, 03°28'W, leg. R. Jocqué & Tanoh, 23.IV.1995 (pitfalls in forest), 1♂ (MRAC 204369); Same data, 7.V.1995, 1♀ (MRAC 204366), 1♀ (MRAC 204368); Same data, 20.V.1995, 1♂ (MRAC 204365), 1♂ (MRAC 204370); Same data, 8.X.1995, 1♂ (MRAC 204372); Same data, 22.X.1995, 1♂ (MRAC 204364); Same data, 5.XI.1995, 1♀ (MRAC 204367), 1imm. 1♂ (MRAC 204371); Same locality, leg. R. Jocqué, 1.XII.1995 (modified Malaise trap), 1♂ (MRAC 200963); Bouaflé, 06°59'N, 05°45'W, 12.I.1981, leg. J. Everts (pitfalls), 3♂ 3♀ (MRAC 174002); Same data, 14.I.1981, 2♂ 1♀ (MRAC 173992); Bouaké, F.-Foro, 07°41'N, 05°02'W, leg. G. Couturier, 3–5.VI.1974 (piège coloré), 1♂ (MRAC 216367); Same data, 15–17.VII.1974, 4♂ (MRAC 216400); Same data, 12–14.VIII.1974, 2♂ (MRAC 216484); Same data, 19–21.VIII.1974, 1♀ (MRAC 216414); Same data, 26–28.VIII.1974, 2♂ (MRAC 216456); Same data, 2–4.IX.1974, 1♂ (MRAC 216433), 1♂ 1♀ (MRAC 216448), 2♂ (MRAC 216420); Bouitha, near Degbézéré, 15km E of Bouaflé, 07°22'N, 06°28'W, leg. R. Schouten & J. Buysen, 21.II.1984, 3imm. 1♂ (MRAC 165970); Gagnoa [06°08'N, 05°56'W], leg. A. Russell-Smith, 30.III.1993 (pitfalls, upland rice), 2♂ 1♀ (PCRS); Mankono, Ranch de la Marahoué, 08°27'N, 06°52'W, leg. J. Everts, I.1980 (riverine forest), 102imm. 40♂ 16♀ (MRAC 172282); Same data, II.1980, 19imm. 58♂ 23♀ (MRAC 172281); Same data, III.1980, 61imm. 106♂ 32♀ (MRAC 172284); Same data, IV.1980, 24imm. 39♂ 34♀ (MRAC 172283); Same data, V.1980, 8♂ 20♀ (MRAC 172280); Same data, VI.1980, 4imm. 2♂ (MRAC 172278); Pakodji, near Degbézéré, 15km E of Bouaflé, 06°59'N, 05°38'W, leg. R. Schouten & J. Buysen, 20.II.1984 (pitfall), 22imm. 19♂ 3♀ (MRAC 165977); Titekro, 20km E of Bouaflé, 06°52'N, 06°20'W, leg. R. Schouten & J. Buysen, 15.II.1984 (pitfalls), 8imm. 10♂ 4♀ (MRAC 165965); Touba [08°16'N, 07°41'W], leg. A. Russell-Smith, 19.VI.1995 (pitfalls, upland rice), 1♂ (PCRS); Warda, Bouaké [07°41'N, 05°01'W], leg. A. Russell-Smith, 7.X.1994 (pitfalls, upland rice), 3♂ 2♀ (PCRS). KENYA: Kakamega Forest, pitfall near quarry, 00°13'N, 34°54'E, 1626m a.s.l., leg. D. Shilabira Smith, 13.XII.2001, 1♂ (MRAC 212708); Same locality, leg. D. Shilabira Smith, 3.I.2001, 1♀ (MRAC 212715); Same locality, 1654m a.s.l., leg. D. Shilabira Smith, 23.II.2002, 1♂ (MRAC 212656); Mathews Range Forest, Near Kitich camp, 01°13'N, 37°18'E, 1339m a.s.l., leg. D. van den Spiegel, 9.XII.2002, 1♂ (MRAC 212743); Mount Kasigau, Jora Village, 03°50'S, 38°39'E, leg. E. Selempo, 1–3.XII.2001, 1♀ (MRAC 213056); Ngaia Forest, 00°19'N, 38°02'E, leg. Jocqué, Warui & Van den Spiegel, 24.IV.2004 (sieved litter), 1♂ (MRAC 215332); Rift Valley Province, Marich Pass Field Studies Centre, 01°32.2'S, 35°27.4'E, leg. W.J. Pulawski & J.S. Schweikert, 26–29.VII.1999, 1♂ (CAS, CASENT 9033277). MALAWI: Chisasira Forest, 25km South of Chintheche, 11°50'S, 33°13'E, leg. R. Jocqué, 1.XII.1977, 1♂ (MRAC 153232); Same data, 20.XII.1977, 3♂ (MRAC 153196); Same data, 3–20.I.1978, 1imm. 1♂ (MRAC 153649); Same locality, leg. R. Jocqué, 3–20.III.1978 (*Brachystegia* woodland), 1♂ 1♀ (MRAC 152985); Nyika plateau, Chelinda [10°35'S, 33°47'E], 2300m a.s.l., leg. R. Jocqué, 7–19.XII.1981 (grassland burned in 1979, pitfalls), 1♂ (MRAC 155686); Same locality, leg. R. Jocqué, 7–19.XII.1981 (grassland burned in 1980, pitfalls), 1♂ (MRAC 155744); Nyika plateau, Chowo rocks [not traced], leg. R. Jocqué, 6–18.XII.1981 (pitfalls in herbaceous vegetation with *Philippia*), 1♂ (MRAC 156302); Same data, 1♀ (MRAC 156384); Nyika plateau, Lake Kaulime [10°34'S, 33°45'E], 2200m a.s.l., leg. R. Jocqué, 6–19.XII.1981 (pitfalls on grassy bank), 2♂ 2♀ (MRAC 155886); Same locality, leg. R. Jocqué, 6–19.XII.1981 (pitfalls on wet bank with *Lobelia*), 1♂ 1♀ (MRAC 156021); Nyika plateau, Manyanjere Forest [not traced], 2100m a.s.l., leg. R. Jocqué, 15.XII.1981 (grassland with stones), 1♀ (MRAC 156722); Nyika plateau, near entrance gate on road Chelinda-Rumphi [not traced], 1700m a.s.l., leg. R. Jocqué, 3–22.XII.1981 (*Brachystegia* woodland, pitfalls), 5♂ 2♀ (MRAC 155822); Same locality, leg. R. Jocqué, 3–22.XII.1981 (secondary *Brachystegia* woodland with *Uapaca*, pitfalls), 1♂ 1♀ (MRAC 155703), 1♂ 1♀ (MRAC 156006), 1♂ 1♀ (MRAC 156062); Same locality, leg. R. Jocqué, 3–22.XII.1981 (pitfalls under large *Brachystegia*), 14♂ 5♀ (MRAC 156289). MOZAMBIQUE: Bartholomew Diaz Point, BD Lodge, 21°15.585'S, 35°06.851'E, 5m a.s.l., leg. C. Haddad, R. Lyle & R. Fourie, 10.XII.2007 (leaf litter, mangroves), 1♂ 3♀ (NCA 2008/194); Bilene, Praia do Bilene, 25°15.649'S, 33°17.659'E, 27m a.s.l., leg. C. Haddad, R. Lyle & R. Fourie, 20.XII.2007 (leaf litter, coastal forest), 1♂ (NCA 2008/210); Chidenguele, Paraiso de Chidenguele, 24°57.276'S, 34°11.860'E, 38m a.s.l., leg. C. Haddad, R. Lyle & R. Fourie, 16.XII.2007 (leaf litter, dune forest), 2imm. 1♂ (NCA 2008/205); Inhaca Island, 26°01'S, 32°54'E, leg. T. Steyn, 6–20.VIII.1994 (beach and dunes, by hand), 1♂ (MRAC 215918); Same locality, leg. T. Steyn, 21.VIII–4.IX.1993 (pitfalls, coastal woodland), 1imm. 4♂ (MRAC 209025); Same data, 18.IX–2.X.1993, 2♂ (MRAC 209418); Same data, 2–16.X.1993, 1♀ (MRAC 209037); Same data, 2–16.X.1993, 2♂ 9♀ (MRAC 209044); Same data, 16–30.X.1993, 7♂ 2♀ (MRAC 208995); Same data, 13–23.XI.1993, 5♂ 5♀ (MRAC 209304); Same data, 27.XI–11.XII.1993, 4imm. 9♂ 1♀ (MRAC 209392); Same data, 25.XII.1993–8.I.1994, 13♂ (MRAC 209400); Same data, 8–22.I.1994, 6♂ 1♀ (MRAC 209468); Same data, 19.II–5.III.1994, 3♂ 3♀ (MRAC 209898); Same data, 5–19.III.1994, 1imm. 4♂ (MRAC 209441); Same data, 19.III–2.IV.1994, 1♂ (MRAC 209732); Same data, 13–30.IV.1994, 1♂ (MRAC 209888); Same data, 30.IV–14.V.1994, 3♂ 1♀ (MRAC 209750); Same data, 14–28.V.1994, 2♂ (MRAC 209774); Same data, 19–25.VI.1994, 2♂ (MRAC 209801); Same data, 9–23.VII.1994, 2♀ (MRAC 209699); Same data, 23.VII–6.VIII.1994, 2♂ (MRAC 209880); Same locality, leg. T. Steyn, 18.IX–2.X.1993, (pitfalls, open parkland), 2imm. 1♂ (MRAC 215982); Same data, 2–16.X.1993, 1♀ (MRAC 209475); Same data, 16–30.X.1993, 5imm. 6♂ 3♀ (MRAC 215999); Same data, 30.X–13.XI.1993, 1♂ (MRAC 209709); Same data, 13–27.XI.1993, 1♀ (MRAC 209414); Same data, 27.XI–11.XI.1993, 2♂ (MRAC 209418); Same data, 11–25.XII.1993, 1♂ (MRAC 209681); Same data, 11–25.XII.1993, 2♂ 2♀ (MRAC 209684); Same data, 25.XII.1993–8.I.1994, 3♂ (MRAC 209693); Same locality, leg. T. Steyn, 15–29.XI.1993 (pitfalls, wetland), 1♀ (MRAC 209325); Same data, 27.XII.1993–10.I.1994, 1♂ (MRAC 209360); Same data, 10–24.I.1994, 1♂ 1♀ (MRAC 209344); Same data, 24.I–7.II.1994, 4♂ (MRAC 208950); Same data, 21.II–5.III.1994, 2♂ (MRAC 209774); Same data, 5–15.III.1994, 2♂ 1♀ (MRAC 209372); Same data, 19.III–2.IV.1994, 4♂ 1♀ (MRAC 209350); Same data, 8–23.IV.1994, 6♂ 2♀ (MRAC 209743); Same data, 23–30.IV.1994, 2♂ 1♀ (MRAC 209786); Same data, 30.IV–14.V.1994, 3♂ 1♀ (MRAC 209903); Same data, 28.V–19.VI.1994, 1♀ (MRAC 209717); Same data, 19–25.VI.1994, 1♂ (MRAC 209796); Same data, 25.VI–9.VII.1994, 1♂ (MRAC 209985); Same data, 20.VIII–3.IX.1994, 1♂ (MRAC 215949); Same data, 3–24.IX.1994, 3♂ 3♀ (MRAC 215922); Same data, 3–24.IX.1994, 3♂ (MRAC 215926); Maxixe [23°52'S, 35°20'E], I.1914, no collector, 1imm. 2♀ (SAMC B6589); Morrungulo, Morrungulo Resort, 23°13.983'S, 35°29.587'E, 12m a.s.l., leg. C. Haddad, R. Lyle & R. Fourie, 6.XII.2007 (leaf litter, dune forest), 1♂ 1♀ (NCA 2008/185); Near Marracuene, Blue Anchor Inn, 25°35.124'S, 32°39.568'E, 50m a.s.l., leg. C. Haddad & R. Fourie, 28.XI.2007 (sifting leaf litter, savanna), 1imm. 2♂ (NCA 2008/165); Near Marracuene, Marracuene Lodge, 25°46.379'S, 32°41.046'E, 12m a.s.l., leg. C. Haddad, 1.XII.2007 (leaf litter, riverine forest), 1imm. 1♀ (NCA 2008/171); Vilankulos, Casa Chibububo, 22°01.231'S, 35°19.237'E, 3m a.s.l., leg. C. Haddad, R. Lyle & R. Fourie, 12.XII.2007 (leaf litter, coastal bush), 1imm. 2♀ (NCA 2008/199); Xai-Xai, Montego's Camp, 25°03.659'S, 33°40.633'E, 28m a.s.l., leg. C. Haddad, 2.XII.2007 (leaf litter, dune forest), 2♀ (NCA 2008/179). NAMIBIA: Caprivi Strip, Popo Falls, 18°07.366'S, 21°34.971'E, leg. R. Lyle, 17.XII.2006 (leaf litter, riverine forest), 1♂ (NCA 2008/4279); Hoarusib River, leg. Museum Expedition, I.1926, 1♀ (SAMC B7110). NIGERIA: Borgu Game Reserve [10°19'N, 03°56'E], leg. A. Russell-Smith, 5–6.V.1973 (flood debris by river bank), 1♀ (BMNH); Ibadan, International Institute of Tropical Agriculture [07°29'N, 03°53'E], leg. A. Russell-Smith, 24.V.1973 (bush fallow), 2♂ 5♀ (BMNH); Same data, VII.1973, 4♂ (BMNH); Same data, 25–29.V.1975, 1♂ (PCRS); Same locality, leg. A. Russell-Smith, 22–26.VI.1973 (cultivated plots), 1♂ 2♀ (BMNH); Iseri [06°30'N, 03°16'E], leg. B. Malkin, 26–30.III.1949, 1♀ (CAS, CASENT 9033106). RWANDA: P.N. Akagera, 50 km north of la pêcherie Ihema, près du lac Mihindi, 01°32'S, 30°43'E, leg. Jocqué, Nsengimana & Michiels, 23.XI–6.XII.1985 (pièges en forêt sèche), 3♂ 2♀ (MRAC 165007); Same locality, leg. Jocqué, Nsengimana & Michiels, 23.XI–6.XII.1985 (bordure de forêt), 1♂ (MRAC 165020); Same locality, leg. Jocqué, Nsengimana & Michiels, 14.XI–3.XII.1985 (Forêt sêche à *Sansevieria*, pièges), 5♂ 1♀ (MRAC 165416). SOUTH AFRICA: *Eastern Cape Province*: Grahamstown [33°18'S, 26°31'E], leg. W.F. Purcell, X.1905, 1♂ (SAMC B7539); Great Fish River at Selbourne, 33°28'S, 27°08'E, leg. M. Burger, 5.XII.1993 (pitfall trap), 1♂ (NCA 96/59); Kentani district [32°30'S, 28°18'E], leg. Abernethy, 1903, 1♂ (SAMC 1289); Mkambathi Nature Reserve, 31°17.364'S, 30°00.284'E, 52m a.s.l., leg. University of KwaZulu-Natal students, 29.I.2008 (pan traps, grassland), 1♂ (NCA 2008/2906); Same locality, 31°15.816'S, 30°02.098'E, 28m a.s.l., leg. Inland Invertebrate Initiative – University of KwaZulu-Natal, 29.I.2008 (pan traps, grassland), 1♀ (NCA 2010/233), 1♀ (NCA 2010/234); Sterkstroom district, Hazelmere Country Lodge, 31°30.126'S, 26°40.815'E, 1542m a.s.l., leg. R. Lyle & R. Fourie, 3–7.XI.2008 (pitfall traps, poplar trees), 1♂ (NCA 2008/4284); Sundays River Valley, 33°23'S, 25°26'E, leg. H. Potgieter, 23.I.1999 (pitfalls in citrus), 3♂ 5♀ (NCA 2000/237); Same data, 23.XI.1999, 3♂ 1♀ (NCA 2000/238). *Free State Province*: Erfenis Dam Nature Reserve, Site 3, *Acacia karroo* trees, 28°30.272'S, 26°47.527'E, leg. R. Fourie & A. Grobler, 30.IX–28.X.2009 (pitfall traps, woodland), 1♂ (NCA 2009/3590); Kroonstad district, Doornkloof farm, 27°43.376'S, 27°42.042'E, leg. R. Fourie & A. Grobler, 29.X–5.XII.2009 (pitfall traps, grassland), 1♂ (NMSA 22690); Mpetsane Conservation Estate, near Clocolan, 28°48'S, 27°39'E, leg. C. Haddad, 9.III.2007 (*Rhus lancea* leaf litter), 1imm. 1♀ (NCA 2008/558); Sandveld Nature Reserve, 27°40'S, 25°41'E, leg. C. Haddad, 25.X.2003 (leaf litter under *Acacia erioloba*), 1♀ (NCA 2002/511); Same data, 25.XI.2003, 1♂ (NCA 2005/77); Tussen-die-Riviere Nature Reserve, 30°29'S, 26°11'E, leg. L. Lotz & C. Haddad, 13.X.2008 (active searching, dense *Acacia* woodland), 1♂ (NMBA 12623). *Gauteng Province*: Balmoral, 25°49.013'S, 28°51.970'E, leg. R. Koko, 11.VII.2006 (pitfall traps), 1♀ (NCA 2008/2782); Buffelsdrift, 25°24.251'S, 28°03.581'E, 1700m a.s.l., leg. R. Koko, I.2006 (incidentals), 1♀ (NCA 2008/2780); Pretoria, Weavind Park, 25°43'S, 28°16'E, leg. C. Anderson, 15.III.1997 (in house), 1♂ (NCA 96/455); Pretoria National Botanical Gardens, 25°44'S, 28°16'E, leg. E. Kassimatis, 6.X–24.XI.2007 (pitfall traps), 1♀ (NCA 2008/1966); Suikerbosrand Nature Reserve, Heidelberg, 26°30.102'S, 28°14.165'E, 1830m a.s.l., leg. H. Roux, 13.XI.2001 (pan trap, grassland plateau), 1♂ (NCA 2008/4278). *KwaZulu-Natal Province*: 15km N of Richard's Bay, 28°40'S, 32°13'E, leg. T. Wassenaar, 5.XII.1995 (rehabilitated coastal forest, sweep net), 1♀ (NCA 96/492); Same locality, leg. T. Wassenaar, 10.XII.1996 (pitfalls, rehabilitated coastal forest), 1♀ (NCA 97/840); Same data, 27.II.1997, 1♂ (NCA 97/842); Botha's Hill [29°43'S, 30°44'E], leg. R.F. Lawrence, XI.1953, 1♀ (NMSA 5951); Cathedral Peak, 28°58.688'S, 29°15.586'E, 1916m a.s.l., leg. Maluti-Drakensburg Transfrontier Park survey, 18.IX.2005 (white pan trap 5, grassland), 1♂ (NCA 2008/1911); Cathedral Peak Forest Station, 75 km WSW of Estcourt [28°56'S, 29°13'E], 1400 m a.s.l., leg. S. & J. Peck, 7–31.XII.1979 (dung traps, veld pasture), 1♂ (AMNH); Drummond [29°45'S, 30°41'E], leg. R.F. Lawrence, XII.1939, 1♂ (NMSA 2633); Empangeni, 28°45'S, 31°54'E, leg. P. Reavell, 1.X.1983 (in pool), 1♀ (NMSA); Enseleni Game Reserve, 13km N Richard's Bay [28°41'S, 31°59'E], leg. P. Reavell, 10.III.1981, 1♀ (NCA 81/198); Garden Castle, 29°44.700'S, 29°12.663'E, 1842m a.s.l., leg. Maluti-Drakensburg Transfrontier Park staff, 2.XI.2005 (white pan trap 5, grassland), 1♂ (NCA 2008/1913); iSimangaliso Wetlands Park, False Bay Park, 27°55'S, 32°16'E, leg. J. Esterhuizen, 13.X.2003 (tsetse fly traps), 1♀ (NCA 2004/769); Same data, 22.X.2003, 1♂ (NCA 2004/765); Same locality, 27°54.014'S, 32°23.543'E, leg. Earthwatch team 9, 15.I.2005 (yellow pan traps, open savanna), 1imm. 1♀ (NCA 2007/1309); Ithala Game Reserve, Near ruins, Ngubhu loop, 27°30.817'S, 31°14.304'E, leg. C. Haddad, 1.VII.2007 (leaf litter), 11imm. 1♂ 1♀ (NCA 2007/2809); Kosi Bay [26°52'S, 32°52'E], leg. R.F. Lawrence, VII.1936, 1♂ (NMSA 158); Same locality, Banga Nek, near third lake, 27°05.134'S, 32°50.533'E, leg. P. & G. Van Niekerk, X. Combrink & J. Warner, 27.II.2007 (sweeps in grass), 1♀ (NCA 2009/4608); Illovo Beach, Mount Edgecombe [30°07'S, 30°51'E], leg. C. Cilliers, 7.I.1977, 1imm. 1♀ (NCA 2007/1137); Mkuzi Game Reserve, 27°40.356'S, 32°15.065'E, leg. Earthwatch Team 1, 18.III.2005 (yellow pan traps, *Terminalia sericea* woodland), 1♂ (NCA 2007/1297); Same locality, 27°35.768'S, 32°14.365'E, leg. Earthwatch Team 10, 22.I.2005 (blue pan traps, *Terminalia sericea* woodland), 1♂ (NCA 2007/1298); Mtunzini, “Twin Streams” Farm (I.F. Garland), 28°57'S, 31°46'E, leg. T. & C. Griswold, P. Croeser & P. Reavell, 19–20.I.1984 (coastal dune forest), 1♀ (NMSA); Natal, no date, leg. Martin?, 1♂ 1♀ (MNHN 6383); Ndumo Game Reserve, Crocodile farm, 26°53'S, 32°19'E, leg. C. Haddad, 8–23.I.2002 (pitfalls), 1♂ (NCA 2002/391); Same locality, Ezikhebeni, Pongola River, 26°53.380'S, 32°19.098'E, leg. C. Haddad, R. Lyle & V. Butler, 28.VI.2009 (leaf litter, riverine forest), 1♀ (TMSA 23612); Same locality, Pongola River floodplain, near pump, Riverine forest, 26°54.323'S, 32°19.435'E, leg. C. Haddad & F. Jordaan, 27.VI.2006 (sieving leaf litter), 1imm. 3♂ 4♀ (NCA 2006/1201); Same locality, Pongola River floodplain, 26°53.384'S, 32°19.097'E, 16.I.2006, leg. C. Haddad (riverine forest leaf litter), 5♂ 1♀ (NCA 2006/710); Same locality, Viewing tower, 26°54.762'S, 32°16.290'E, leg. C. Haddad, R. Lyle & V. Butler, 30.VI.2009 (leaf litter, broadleaf woodland), 2♂ 2♀ (TMSA 23564); Same locality, Western shore of Shokwe Pan, 26°50'S, 32°12'E, leg. C. Haddad, 3.VII.2002 (leaf litter, *Ficus sycomorus* forest), 1♂ (NCA 2002/392); Same locality, Western shore of Shokwe Pan, 26°52.418'S, 32°12.590'E, leg. C. Haddad, R. Lyle & V. Butler, 8.VII.2009 (leaf litter, *Ficus* forest), 1♀ (TMSA 23548); Near Port Shepstone [30°45'S, 30°26'E], leg. W.F. Purcell, IX.1905, 1♀ (SAMC 150751); Ngome State Forest, 27°49'S, 31°26'E, leg. M. van der Merwe, XI.1992 (pitfalls, open forest), 1♀ (NCA 94/396); Same locality, leg. M. van der Merwe, XII.1992 (pitfalls, grass), 1♂ (NCA 94/475); Same data, I.1993, 1♂ (NCA 94/441); Ophathe Game Reserve, Ophathe River bed, 28°22.693'S, 31°24.442'E, leg. C. Haddad & R. Fourie, 5.VII.2007 (leaf litter, river bank), 4imm. 2♂ (NCA 2007/2969); Same locality, Montane grassland, 28°25.344'S, 31°23.957'E, 897m a.s.l., leg. C. Haddad, 4.X.2008 (sifting leaf litter), 1imm. 1♀ (NCA 2008/3910); Same locality, Ophathe River Bed, 28°23.727'S, 31°23.643'E, 455m a.s.l., leg. C. Haddad, 30.IX–4.X.2008 (pitfall traps), 2♂ (NCA 2008/4245); Same locality, leg. C. Haddad, 2.X.2008 (active searching), 1♂ 1♀ (NCA 2008/4222); Same locality, Rocky mountainside, 28°23.202'S, 31°24.077'E, 505m a.s.l., leg. C. Haddad, 1.X.2008 (active searching), 1♀ (NCA 2008/4068); Same locality, leg. C. Haddad, 1.X.2008 (sifting leaf litter), 3imm. 1♂ (NCA 2008/4039); Pietermaritzburg [29°37'S, 30°23'E], leg. P. Croeser, 7.XII.1983 (dense fern in garden), 1♀ (NMSA 18487); Same locality, Town Bush Valley, Southern slopes of Hogsback Mountain, 29°33'S, 30°21'E, 3200–3400ft a.s.l., leg. C. Griswold & T. Meikle-Griswold, 11.XI.1984 (weedy vegetation), 1imm. 1♂ 1♀ (NMSA); Sani Pass, 29°39.022'S, 29°27.047'E, 1500m a.s.l., leg. D. Prentice, IX.2009 (pitfall traps, 6d), 1♂ (NCA 2010/271); Same locality, 29°37.217'S, 29°23.330'E, 1800m a.s.l., leg. D. Prentice, IX.2009 (pitfall traps, 5d), 1♂ (NCA 2010/272); Same locality, 29°36.205'S, 29°18.753'E, 2400m a.s.l., leg. D. Prentice, IX.2009 (pitfall traps, 3c), 1♂ (NCA 2010/221); Scottburgh [30°17'S, 30°45'E], leg. W.G. Rump, II.1943, 1♀ (NMSA 3882); Sodwana Bay, 27°24'S, 32°45'E, leg. R. Harris, XI.1982, 1♀ (NCA 83/247); Tembe Elephant Park, 27°01'S, 32°24'E, leg. C. Haddad, 5.I.2002 (leaf litter, deep sand forest), 1imm. 1♂ (NCA 2002/396); Same locality, 27°01'S, 32°24'E, leg. C. Haddad, 3–23.I.2002 (pitfalls, deep sand forest), 1imm. 7♂ 1♀ (NCA 2002/393); Same locality, 26°57'S, 32°26'E, 3–23.I.2002, leg. C. Haddad (pitfalls, closed woodland/clay), 1♂ (NCA 2002/394); Same locality, near offices, 27°03'S, 32°25'E, leg. C. Haddad, 3–23.I.2002 (pitfalls, open woodland/sand), 4♂ (NCA 2002/395); Same locality, 27°03'S, 32°25'E, leg. C. Haddad, 8.II.2005 (sifting leaf litter, open woodland/sand), 3♀ (NCA 2007/3606); Vernon Crookes Nature Reserve, camp, 30°16'S, 30°37'E, leg. L. Lotz, 27.IX.1995, 1♂ (NMBA 7719). *Limpopo Province*: Kruger National Park, Maduringwe, 22°35'S, 31°09'E, leg. R.F. Lawrence, 20.XII.1962, 1♂ (NMSA); Lajuma Mountain Retreat, 23°02'S, 29°27'E, leg. N. Schönhofer, 9.X.2002 (hand collecting), 1♂ (NCA 2007/1153); Same locality, Island 3, 23°01.890'S, 29°26.167'E, leg. M. Mafadza, 23.XI.2004 (sifting leaf litter), 1♀ (NCA 2005/1882); Same locality, Short Forest 3, 23°02.165'S, 29°26.985'E, leg. M. Mafadza, 28.XI.2004 (pitfall trap), 1♀ (NCA 2005/2021); Same locality, Tall forest 3a, 23°02.229'S, 29°26.717'E, leg. M. Mafadza, 28.XI.2004 (pitfall trap), 1♂ (NCA 2005/2022); Same locality, Woodland 3, 23°02.532'S, 29°26.897'E, leg. M. Mafadza, 6.XII.2004 (active search), 1♀ (NCA 2005/1881); Same locality, Woodland 5c, 23°02.528'S, 29°26.866'E, leg. M. Mafadza, 28.XI.2004 (pitfall trap), 1♂ 1♀ (NCA 2005/2023); Little Leigh, 22°56.910'S, 29°52.177'E, 1084m a.s.l., leg. F. Mbedzi, 22.XI.2005 (leaf litter, gallery forest), 1♀ (NCA 2008/2764), 1♂ (NCA 2008/2765); Marble Hall, Schoeman Boerdery, 24°57'S, 29°17'E, leg. P. Stephen, 16.XI.1999 (pitfalls in citrus), 1♂ (NCA 2000/204); Nylsvley Nature Reserve, 24°39'S, 28°40'E, leg. C. Schultz, 1.XII.1975, 1♂ (NCA 2007/1154); Springbokvlakte, Settlers (wildskamp), 24°54'S, 28°43'E, leg. M. van Jaarsveld, 9.I.2002 (pitfalls, grassland), 1♀ (NCA 2003/1328). *Mpumulanga Province*: 20km NE of Brondal, 25°21'S, 30°50'E, leg. M. van den Berg, 16.IX.1997 (on Hass avocados), 1imm. 1♂ (NCA 98/196); Same data, 2.XII.1997, 1♂ (NCA 98/197); Same locality, leg. M. van den Berg, 16.IX.1997 (on Fuerte avocados), 1♂ (NCA 98/198); Groblers Farm, 25°29'S, 30°05'E, leg. L. Makaka, 29.XI–2.XII.2008 (pitfall traps, grassland AF2), 1♂ (NCA 2010/265); Same locality, leg. L. Makaka, 26–29.XI.2008 (pitfall traps, grassland AF3), 1♂ (NCA 2010/229); Same data, 29.XI–2.XII.2008, 1♂ (NCA 2010/227), 1♂ (NCA 2010/266); Same locality, 26–29.XI.2008, leg. L. Makaka (pitfall traps, grassland AF4), 2♂ (NCA 2010/228), 1♂ (NCA 2010/267), 1♂ (NCA 2010/268); Guernsey Farm, 15km NW of Klaserie [24°03'S, 31°12'E], leg. S. & J. Peck, 19–31.XII.1985 (Malaise traps, woodland), 2♂ (AMNH); Hall and Sons, 10km NE of Nelspruit, 25°21'S, 31°46'E, leg. M. van den Berg, 21.VII.1997 (on Hass avocados), 6imm. 2♂ 3♀ (NCA 98/216); Same data, 10.III.1998, 3imm. 1♂ (NCA 98/1065); Same locality, leg. M. van den Berg, 23.X.1997 (on Fuerte avocados), 1imm. 3♀ (NCA 98/217); Same data, 12.XII.1997, 1♂ (NCA 98/776); Hectorspruit, Vergelegen, 25°25'S, 31°40'E, leg. P. Stephen, 12.X.1998 (pitfalls in citrus), 1imm. 1♀ (NCA 99/193); Nelspruit, 25°21'S, 31°46'E, leg. M. van den Berg, 9.XII.1997 (on macadamia nuts), 1♀ (NCA 98/829); Nelspruit, Institute for Tropical and Subtropical Crops, Waaierproef, 25°21'S, 31°46'E, leg. M. van den Berg, 18.XI.1997 (on macadamia tree), 1imm. 1♀ (NCA 98/174); Same data, 9.XII.1997, 1♀ (NCA 98/829); Same data, 12.II.1998, 1♂ (NCA 98/830); Nelspruit Agricultural College, 25°21'S, 31°46'E, leg. P. Stephen, 12.XI.1999 (pitfalls in citrus), 1♀ (NCA 2000/185); Nelspruit Nature Reserve [25°30'S, 30°58'E], leg. Endrody-Younga, 23.XI.1986, 1♂ (TMSA 19679); Roger Croall, 25°33'S, 30°05'E, leg. L. Makaka, 26–29.XI.2008 (pitfall traps, grassland R1), 1♂ (NCA 2010/269); Same locality, leg. L. Makaka, 26–29.XI.2008 (pitfall traps, grassland R4), 1♂ (NCA 2010/263), 1♂ (NCA 2010/264); Sakhelwe location, 25°24'S, 30°05'E, leg. L. Makaka, 26–29.XI.2008 (pitfall traps, grassland COM3), 1♂ (NCA 2010/224); Veloren Vallei Nature Reserve, Block 3, 25°18.832'S, 30°07.791'E, leg. L. Makaka, 4–7.III.2009 (pitfall traps, grassland V3.4), 1♀ (NCA 2010/225); Witbank Dam Nature Reserve, 25°51'S, 29°18'E, leg. A. Leroy, 9.XI.1991 (grassland), 1♀ (NCA 92/172). *North West Province*: Matshaneng district, Hermitage Farm, 27°04.136'S, 23°40.991'E, leg. C. Haddad, 1.XII.2003–22.I.2004 (pitfalls under trees), 1♀ (NCA 2005/2012); Potchefstroom district, Thabela Thabeng Mountain Retreat, 26°51.825'S, 28°17.819'E, leg. R. Fourie & A. Grobler, 1–29.X.2009 (pitfall traps, woodland grassland), 10♂ (NCA 2009/3553); Same locality, 26°51.828'S, 28°17.805'E, leg. R. Fourie & A. Grobler, 1–29.X.2009 (pitfalls, Vaal River bank), 4♂ 1♀ (NCA 2009/3561). *Northern Cape Province*: Prieska district, Green Valley Nuts, 29°35'S, 22°56'E, leg. C. Haddad, 19.XII.2001 (fogging, pistachio tree canopy), 1♂ (NCA 2002/481); Same locality, 22°56.683'S, 29°35.184'E, leg. C. Haddad, 23.XI–18.XII.2001 (pitfalls, *Eucalyptus* trees), 2♂ 1♀ (NCA 2006/1289); Kuruman district, Sunnyside Farm, 27°43.514'S, 23°36.812'E, leg. C. Haddad, 1.XII.2003–22.I.2004 (pitfalls, gravel bed), 1♀ (NCA 2005/2013). *Western Cape Province*: Brenton-on-Sea, 34°04'S, 23°02'E, leg. H.G. Robertson, 1–7.XII.1996 (pitfall traps, broken fynbos), 1♂ (SAMC ENW-C005376); De Hoop Nature Reserve, Bitou number 2, 34°27.194'S, 20°24.250'E, leg. C. Haddad & R. Lyle, 25.IX.2007 (sifting leaf litter), 2♂ 1♀ (NCA 2007/3896); Same locality, Potberg, 34°22.549'S, 20°32.004'E, leg. C. Haddad, 4.IV.2004 (sieving leaf litter), 16♂ 1♀ (NCA 2008/576); Knysna, Uitzicht Annex, 34°00'S, 23°20'E, leg. L. Lotz, 13–19.X.1998 (pitfall trap), 3♂ (NMBA 7420). TANZANIA: *Coast Region*: Kisarawe District, Kazimzumbwe Forest Reserve, 06°57'S, 39°03'E, leg. Frontier Tanzania, I–II.1991, 1♂ (ZMUC), 1♂ (ZMUC), 1♂ (ZMUC), 7♂ 5♀ (ZMUC), 2♂ (ZMUC), 5♂ 2♀ (ZMUC); Same locality, leg. Frontier Tanzania, I–II.1992, 1♂ (ZMUC); Rufigi District, Namakutwa Forest Reserve, 08°19'S, 39°00'E, leg. Frontier Tanzania, VIII–IX.1992, 19♂ 5♀ (ZMUC). *Iringa Region*: Uzungwa Mountains, Uzungwa Scarp Forest Reserve, above Chita Village [08°20'S, 35°56'E, 1500m a.s.l., leg. N. Scharff, 2–13.XI.1984 (pitfall traps, montane rain forest), 2♂ (ZMUC); Same locality, leg. N. Scharff, 25–29.X.1984 (pitfall traps, lowland rain forest), 1♀ (ZMUC). *Kilimanjaro Region*: Mkomazi Game Reserve, Ibaya camp, 03°58'S, 37°48'E, leg. A. Russell-Smith, 19–20.XI.1994 (pitfalls, unburnt grassland), 4♂ 6♀ (MRAC 211327). *Lindi Region*: Lindi District, Litipo Forest Reserve, 10°02'S, 39°29'E, leg. Frontier Tanzania, VII–IX.1993, 17♂ 9♀ (ZMUC), 21♂ 10♀ (ZMUC), 20♂ 8♀ (ZMUC). *Mbeya Region*: 8km NE of Kyela, 09°35'S, 33°48'E, leg. R. Jocqué, 10–19.XI.1991 (pitfalls in miombo relict), 4♂ 4♀ (MRAC 173 920); Itungi, 09°36'S, 33°55'E, leg. R. Jocqué, 10.XI–1.XIII.1991 (pitfalls in swamp with floating vegetation, edge high reeds), 1♂ (MRAC 173940), 1♂ (MRAC 173960); Matema, 1km N of Livingstone mountains, 09°30'S, 34°03'E, leg. R. Jocqué, 14–24.XI.1991 (pitfalls, evergreen forest), 2♂ (MRAC 173204). *Morogoro Region*: 62 road km SW of Morogoro, 07°02.5'S, 37°15.3'E, leg. W.J. Pulawski, 2.I.2003, 1♀ (CAS); Morogoro District, Kimboza Forest Reserve, 07°01'S, 37°48'E, leg. Frontier Tanzania, I–III.1994, 9♂ 8♀ (ZMUC); Mwanihana Forest Reserve, 700m a.s.l., leg. N. Scharff, 8–16.IX.1984 (pitfall traps, lowland rain forest), 1♂ (ZMUC), 1♂ (ZMUC), 1♂ (ZMUC). *Pwani Region*: Bagamoyo District, Sadani Zaraninge Forest Reserve, 06°10'S, 38°39'E, leg. Frontier Tanzania, VII–VIII.1991 (pitfalls, dry coastal forest), 1♂ (ZMUC). *Tanga Region*: Mbomole Hill, 05°05.7'S, 38°37'E, 1000m a.s.l., leg. C.E. Griswold, N. Scharff & D. Ubick, 5–8.XI.1995, 1♂ (CAS, CASENT 9033142); Muheza District, Magrotto Hill, 05°07'S, 38°45'E, leg. Frontier Tanzania, VII–IX.1994, 3♂ 1♀ (ZMUC), 2♂ 2♀ (ZMUC); Muheza District, Manga Forest Reserve, 05°02'S, 34°47'E, leg. Frontier Tanzania, VIII.1997, 1♂ (ZMUC); Muheza District, Pangani Falls Forests, 05°20'S, 38°40'E, leg. Frontier Tanzania, I–III.1993 (riverine and dry forest), 8♂ (ZMUC). TOGO: Bassari, 09°15'N, 00°47'E, leg. P. Douben, V–VII.1994 (pitfalls), 1♂ 1♀ (MRAC 173991); Bassari, Entre Bassari et Sokode, 09°15'N, 00°47'E, leg. P. Douben, V–VII.1984 (savanna boisée), 4♂ 2♀ (MRAC 166237), 1♀ (MRAC 166176); Dzobégan, 07°14'N, 00°41'E, leg. S. Tchibozo, I.2003 (in house), 1♂ (MRAC 212776). UGANDA: Entebbe [00°04'N, 32°27'E], leg. P.L.G. Benoit, 1959, 1♂ (MRAC 131303); Kampala, Namulonge Research Station [00°32'N, 32°35'E], leg. A. Russell-Smith, 22.IV.1994 (in maize field), 1♂ (PCRS); Kanyawara, 00°34'S, 30°21'E, 1600m a.s.l., leg. V. & B. Roth, 30.X.1992, 1♂ (CAS, CASENT 9033135). ZAMBIA: Between Namwala and Lake Itezhi-Tezhi, Pontoon road, 15°41.887'S, 26°21.588'E, leg. C. Haddad, 5.XII.2006 (leaf litter), 1imm. 1♀ (NCA 2007/900); Kafue National Park, Near Namwala, Chibila Camp, 15°46.636'S, 26°00.405'E, leg. C. Haddad & J. Parau, 7.XII.2006 (leaf litter), 1♂ (NCA 2007/576); Kasanka National Park, Fibwe Camp, 12°33'`S, 30°13'E, leg. C. Stuart, 15.II.2001, 1♀ (NCA 2002/540); Same data, 11.XI.2001, 1♀ (NCA 2002/550); Livingstone, Quarry nr Livingstone Airport, 17°47.998'S, 25°46.588'E, leg. C. Haddad & J. Parau, 1.XII.2006 (leaf litter), 3♀ (NCA 2007/624); Near Mpulungu, 08°48'S, 31°05'E, leg. W.J. Pulawski, 20.III.1998, 1♀ (CAS, CASENT 9033105); Wildlives Game Farm, near Choma, Hunter's Camp, 16°58.957'S, 26°36.973'E, leg. C. Haddad, J. Parau & F. Jordaan, 3.XII.2006 (leaf litter), 1♂ 7♀ (NCA 2007/470); Same locality, Open savanna, 16°58.974'S, 26°38.974'E, leg. C. Haddad, 4.XII.2006 (leaf litter), 1♂ (NCA 2007/553); Same locality, Siatichema River, 16°59.615'S, 26°38.093'E, leg. C. Haddad, 3.XII.2006 (leaf litter), 4♀ (NCA 2007/1128). ZIMBABWE: Bulawayo, Hillside, 20°10'S, 28°35'E, leg. M. FitzPatrick, II.1999 (pitfalls), 1♀ (NMZA 13854); Harare, 6km NW of Westwood HQ, Girls College [17°49'S, 30°59'E], leg. Natural History Museum of Zimbabwe staff, 7.XII.1993 (under logs), 2♂ (NMZA 11157); Victoria Falls, 17°56'S, 25°50'E, leg. W.J. Pulawski, 1–8.II.1995, 1♂ (CAS, CASENT 9033083).

**Figures 67–70.**
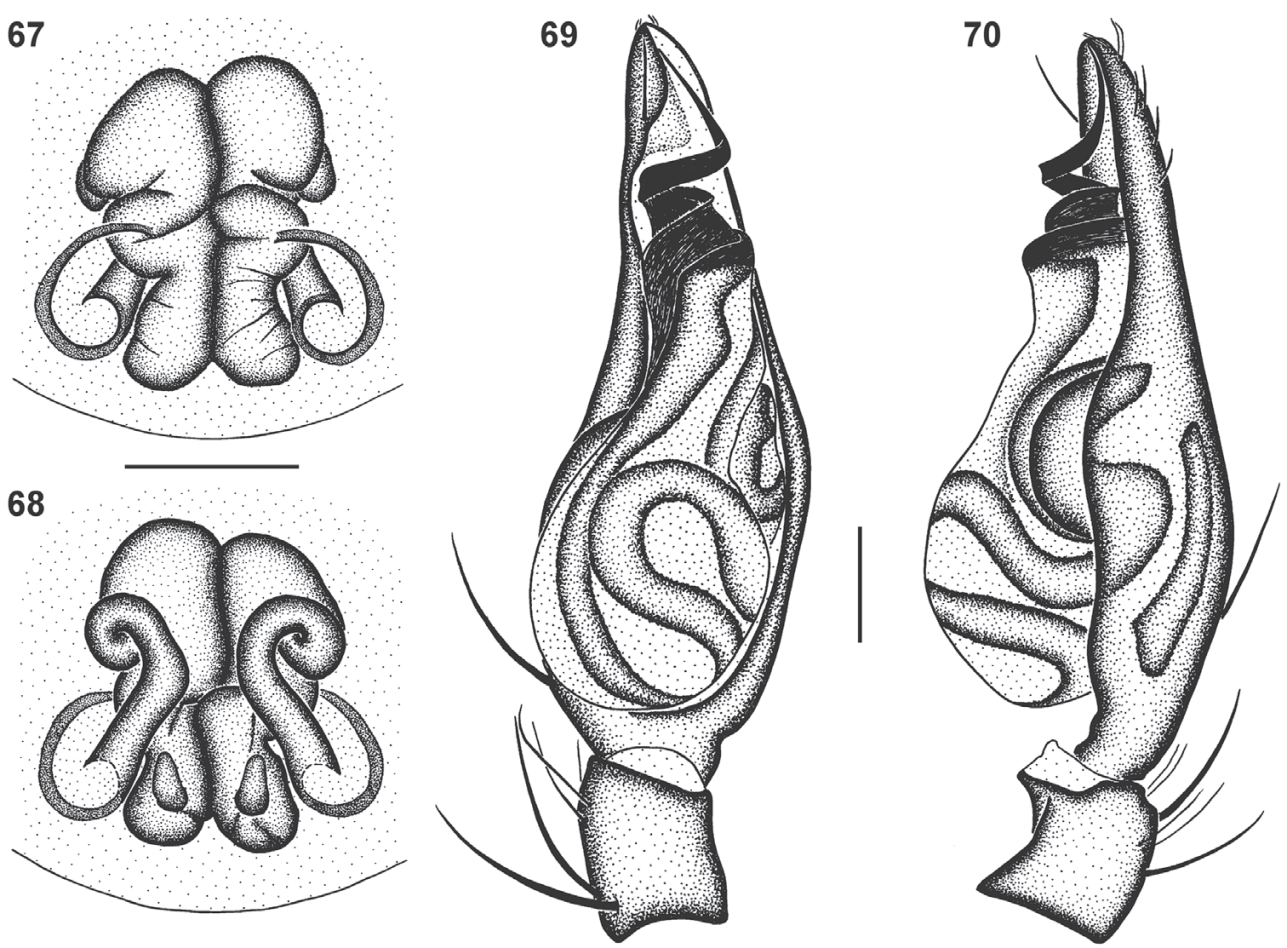
Genitalic morphology of *Copa flavoplumosa* Simon, 1885: **67** female epigyne, ventral view **68** same, dorsal view **69** male palp, ventral view **70** same, retrolateral view. Scale bars = 0.25 mm.

**Figure 71.**
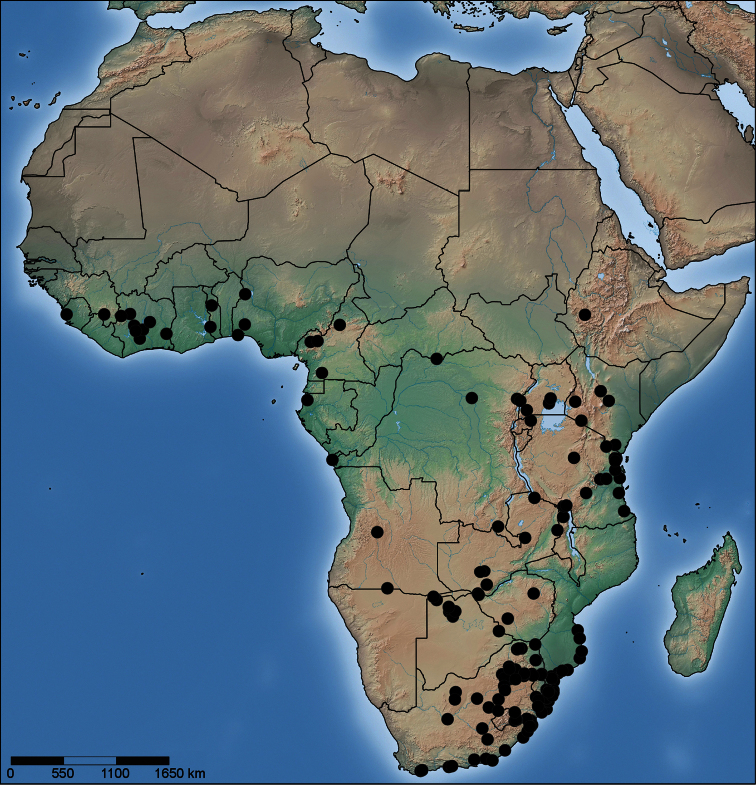
Distribution of *Copa flavoplumosa* Simon, 1885 in the Afrotropical Region.

##### Diagnosis.

*Copa flavoplumosa* is a distinctive species, easily recognisable from congeners by the large 6-shaped epigynal ridges and long copulatory ducts with an anterior loop in the females, and males by the embolus with a broad base and long, slightly curved distal section.

##### Remarks.

The type locality of *Copa flavoplumosa* is given by Simon (1885) as Congo: Landana. This locality is within the modern Angolan enclave of Cabinda that is surrounded by the D.R. Congo. Although the syntype series includes a male, this sex was not originally described by Simon (1885), and this specimen is therefore not designated as a paralectotype. A lectotype female and paralectotype female are designated here, of which the larger of the two in the type series is the lectotype.

The specimens of *Copa benina nigra* Lessert, 1933 available in the MNHG are not specifically labelled as types and their status is thus uncertain, although the labels indicate localities consistent with that in Lessert's (1933) description, i.e. Chimporo and Rio Mbale. Neither of these localities could be traced on modern maps or electronic resources. Some maps from the early 20^th^ century indicate that the Rio Mbale runs northwards between 16°20'E and 16°40'E with its source at approximately 12°00'S in central Angola. Chimporo has been cited by Mansell (1996) as being located at 17°20'S, 17°17'E in southern Angola. From Lessert's (1933) figure of the male embolus it is clear that *Copa benina nigra* is a junior synonym of *Copa flavoplumosa*.

##### Female

**(Parc National Albert, MRAC 234182).** Measurements: CL 3.60, CW 2.69, AL 4.85, AW 3.10, TL 8.20 (6.35–9.30), FL 0.40, SL 1.70, SW 1.58, AME–AME 0.10, AME–ALE 0.02, ALE–ALE 0.44, PME–PME 0.12, PME–PLE 0.13, PLE–PLE 0.56, PERW 0.78, MOQAW 0.40, MOQPW 0.43, MOQL 0.57.

Length of leg segments: I 2.60 + 1.23 + 2.04 + 2.05 + 1.20 = 9.12; II 2.58 + 1.20 + 1.93 + 2.03 + 1.18 = 8.85;III 2.55 + 1.16 + 1.89 + 2.25 + 1.25 = 9.10;IV 3.08 + 1.30 + 1.98 + 3.56 + 1.38 = 11.30.

General appearance as in Fig. 7. Carapace bright yellow-orange, eye region black; broad median black line comprising black feathery setae from PER to posterior slope of carapace, broken up by narrow asetose line from between PME to midpoint and Y-shaped asetose area from fovea towards anterior coxae and posterior of carapace; striae absent; lateral margins with narrow fringe of black feathery setae; areas between markings covered in orange feathery setae. All eyes with black rings; AER procurved, medians much larger than laterals; AME separated by distance equal to ½ their diameter; AME separated from ALE by distance approximately ^1^∕_10_ AME diameter; clypeus height slightly less than 1½ AME diameter; PER strongly procurved, eyes subequal in size; PME separated by distance equal to ¾ their diameter; PME separated from PLE by distance equal to ^4^∕_5_ PME diameter; CW:PERW = 3.45:1. Chelicerae yellow-orange, with pectinate curved setae on promargin; promargin with two teeth separated by basal width of proximal tooth, distal tooth much larger; retromargin with two teeth separated by ½ their basal width, distal tooth slightly larger than proximal tooth, close to fang base. Endites yellow, cream prolaterally and distally, with small black prolateral proximal markings; labium yellow-orange, cream distally, without markings; sternum pale orange, without markings (Fig. 9). Legs yellow-brown, posteriors slightly darker, femora slightly darker dorsally than ventrally; femora with broad dorsal line between proximal and distal spines and incomplete dorsal rings at ⅔ their length and distally, each composed of black feathery setae; patellae with dense black feathery setae laterally; tibiae I & II without markings, with scattered black feathery setae, III & IV with rings proximally and medially corresponding to ventral spines, distal ends with black ring, all covered in black feathery setae with white feathery setae between them; metatarsi I & II without markings, with scattered black and white feathery setae, III & IV with proximal, medial and distal rings corresponding to paired leg spines, covered in black feathery setae with white feathery setae between them; tarsi uniform yellow; palp yellow, spines without spots. Leg spination: femora: I pl 2 do 3 rl 2, II pl 2 do 3 rl 2, III pl 2 do 3 rl 1-2, IV pl 2 do 3 rl 1; all femora with scattered erect ventral setae; patellae: I & II with fine proximal and distal do setae, III & IV with proximal and distal do spines, proximal spine finer and shorter than distal; tibiae: I do 1 pl 2 long fine setae, plv 2 rlv 2 spines, II do 1 long fine seta, pl 2 plv 2 rlv 1 spines, III pl 2 do 1 rl 2 plv 2 rlv 2 vt 2, IV pl 2 do 1 rl 2 plv 2 rlv 1 vt 2; metatarsi: I plv 2 rlv 2, II plv 2 rlv 2, III pl 3 rl 3 plv 2 rlv 2 vt 3, IV pl 3 rl 3 plv 2 rlv 2 vt 3. Palpal spination: femora: pl 1 do 2 rl 1, with scattered erect ventral setae, mainly retrolaterally; patellae: pl 1 do 2; tibiae: pl 1 do 2 plv 1-2; tarsi: pl 1 rl 1 plv 2 rlv 1. Abdomen with very small orange-brown anterior dorsal scutum; dorsum cream, densely covered in black straight and feathery setae, interspersed with white feathery setae forming small spots in anterior two-thirds and fine transverse chevrons posteriorly; sides of abdomen cream, densely covered in white feathery setae; venter cream, covered in short straight black setae, with broad densely setose subrectangular marking medially from epigastric furrow to spinnerets, comprising black and white feathery setae and short straight black setae. Epigyne longer than broad, with large 6-shaped ridges laterally at midpoint of epigyne, separated by approximately 1½ times their width, with copulatory openings distinct (Figs 55, 67); copulatory ducts directed anteriorly, slightly obliquely, with anterior bend and characteristic loop before entering anterior ST II; broad ducts connecting ST II to elongate posterior ST I; ST I clearly narrower than ST II (Fig. 68).

##### Male

**(Mikembo, MRAC 234447).** Measurements: CL 3.30, CW 2.55, AL 3.55, AW 2.00, TL 6.60 (5.20–8.90), FL 0.37, SL 1.43, SW 1.41, AME–AME 0.06, AME–ALE 0.02, ALE–ALE 0.38, PME–PME 0.10, PME–PLE 0.11, PLE–PLE 0.49, PERW 0.68, MOQAW 0.37, MOQPW 0.37, MOQL 0.50.

Length of leg segments: I 2.28 + 1.08 + 1.90 + 1.95 + 1.23 = 8.44; II 2.23 + 1.06 + 1.78 + 1.93 + 1.18 = 8.18; III 2.20 + 1.05 + 1.80 + 2.20 + 1.20 = 8.45; IV 2.95 + 1.20 + 2.25 + 3.32 + 1.33 = 11.05.

General appearance as in Fig. 8, male more slender than female. Carapace bright orange, markings and setae as for female. All eyes with black rings; AER procurved, medians much larger than laterals; AME separated by distance equal to ^2^∕_5_ their diameter; AME separated from ALE by distance approximately ^1^∕_10_ AME diameter; clypeus height slightly larger than double AME diameter; PER strongly procurved, medians very slightly larger than laterals; PME separated by distance slightly less than ⅔ their diameter; PME separated from PLE by distance slightly larger than ^4^∕_5_ PME diameter; CW:PERW = 3.75:1. Chelicerae orange, with curved setae on promargin not pectinate; dentition as for female. Endites, labium and sternum as for female. Legs yellow-brown, posteriors slightly brighter yellow and darker, markings as for female. Leg spination: femora: I pl 2-3 do 3 rl 1-2, II pl 2 do 3 rl 2, III pl 2 do 3 rl 2, IV pl 2 do 3 rl 2; all femora with scattered erect ventral setae; patellae: I & II with fine proximal and distal do setae, III & IV with proximal and distal do spines, proximal spine finer and shorter than distal; tibiae: I pl 1 do 1 rl 1 long fine setae, plv 2 rlv 2 spines, II do 1 rl 1 long fine setae, pl 2 plv 2 rlv 2, III pl 2 do 1 rl 2 plv 2 rlv 2 vt 2, IV pl 2 do 1 rl 2 plv 2 rlv 2 vt 2; metatarsi: I plv 2 rlv 2, II plv 2 rlv 2, III pl 3 rl 3 plv 2 rlv 2 vt 3, IV pl 3 rl 3 plv 2 rlv 2 vt 3. Palpal spination: femora: pl 1 do 2 rl 1, with scattered erect ventral setae; patellae: pl 1 do 2; tibiae: pl 1 do 1 plv 1; tarsi: pl 1 plv 2. Abdomen with orange-brown dorsal scutum extending just past midpoint; dorsum cream, densely covered in black straight and feathery setae with scattered white feathery setae, with patches of white feathery setae forming small spots in anterior two-thirds and fine transverse chevrons posteriorly; sides of abdomen cream, densely covered in white feathery setae with scattered yellow-orange feathery setae; venter cream, covered in short straight black setae, with broad densely setose marking medially from epigastric furrow, converging at spinnerets, comprising black and white feathery setae and short straight black setae. Male palpal cymbium orange-brown, with several thicker bent setae distally (Fig. 70); tegulum pear-shaped, dark red-brown, with nearly black ducts; embolus with broad oblique base directed prolaterally and distally, proximal coil broad and nearly transverse, distal section slightly curved and variable in length (Figs 56, 61–65, 69).

**Colour variation.** Throughout the geographical range of *Copa flavoplumosa* there is considerable variation in the colouration of specimens, particularly with regard to the intensity of yellow/orange and white/cream markings on the body. Three main generalised colour forms can be found. The most widespread variation has a yellow-brown to bright orange carapace and abdomen, with black markings (Fig. 1), and is found throughout the region except in the rainforests of central Africa. This colour form is mainly associated with populations in savanna and grassland habitats.

The second colour form (corresponding to the redescriptions above) has a yellow to orange carapace with black markings and a black abdomen with white markings, similar in pattern and arrangement to the previous form (Figs 2, 3, 7, 8). This form is found in moist savannas and forests across tropical Africa. While most South African populations of this species have colouration corresponding to the first form described here, the populations in the fynbos and grasslands along the southern coast of the country also have a black abdomen with white markings, but the carapace is even darker, nearly dark red-brown in colour.

The third form, corresponding to the description of *Copa benina nigra*, is a nigrito form restricted to central and western Africa but only occurring in isolated populations. This form has an entirely black body with white markings corresponding to those described for the other two types above (Fig. 4).

The distribution of the three forms can partly be explained by the habitats they occupy, although some populations (e.g. Faro Game Reserve in Cameroon and Mankono in Ivory Coast) have representatives of all three colour forms but in varying proportions. The colouration of the first form is clearly an adaptation for camouflage in the litter layer of savanna and other habitats that are exposed to sunlight for a considerable portion of the day. The second form apparently occurs in closed canopy forests and dense woodlands that are shaded for most of the day or the entire day. The black abdominal colouration with strongly contrasting markings enables these spiders to blend into dark patches with low light levels in these habitats. The nigrito form can exploit such microhabitats in a similar way, but this does not explain the occurrence of this colour form at some Miombo woodland localities (e.g. Wildlives Game Farm in Zambia). A possible explanation for this case could be the occurrence of natural fires in these habitats. The burned trees, logs and grasses in such disturbed environments may provide sites where these spiders may optimally exploit their colouration for camouflage. Whether individuals have the capability for colour change in response to changing environmental conditions (e.g. following fire) through the use of chromatophores or ommochromes, or whether individual phenotypes are stable, has yet to be determined and should be the subject of future research.

**Distribution.** Widespread throughout the continental Afrotropical Region (Fig. 71).

##### Biology.

Specimens were mainly collected from the leaf litter layer of all of the main biome types in Africa except for true deserts and karoo habitats, although records from semi-arid temperate grasslands and dry savannas are scarce. The greatest density of records is in moist savannas and closed canopy forests, although the species seems largely absent from rainforests; considering the extensive sampling in the D.R. Congo, especially by the MRAC, only three records from rainforests in this country are reported here. Specimens were most regularly collected by pitfall traps, litter sifting and by hand from the ground surface.

#### Copa kei sp. n.

urn:lsid:zoobank.org:act:A4CC7016-5E90-4687-8936-2F07FE3BDE0F

http://species-id.net/wiki/Copa_kei

Figures 5, 6 10–12 19 –30 49–54 57–60 66 72–75 76

##### Type material.

**Holotype female.** SOUTH AFRICA: *Eastern Cape Province*: Kei Mouth, 32°41.206'S, 28°22.497'E, leg. C. Haddad, 25.IX.2004 (grass at tree base) (NCA 2007/3843).

##### Paratypes.

SOUTH AFRICA: *Eastern Cape Province*: Cwebe Nature Reserve, The Haven, 32°14.497'S, 28°54.653'E, leg. C. Haddad, 30.X.2006 (grassy litter behind dunes), 1♂ (NCA 2008/270); Dwesa Nature Reserve, 32°16.2'S, 26°52.2'E, leg. M. Mgobozi, X.2004 (pitfall traps), 2♂ 2♀ (NCA 2008/1967); East London, Pineapple Research Station, 33°00.6'S, 26°54.0'E, leg. D. Keetch, 15.III.1985 (on soil, coastal dune forest), 1♂ 5♀ (NCA 95/325); Hogsback, Never Daunted Lodge, 32°35.729'S, 26°55.894'E, 1250m a.s.l., leg. C. Haddad, 7.I.2011 (night collecting), 1♀ (NCA 2010/2750); Same locality, Tyume Forest, near Big Tree, 32°36.123'S, 26°56.687'E, 1070m a.s.l., leg. C. Haddad, 28.III.2011 (sifting litter, Afromontane forest), 1♂ (TMSA 24012); Katberg, Katberg Pass, 32°28.710'S, 26°40.337'E, leg. J.A. Neethling & C. Luwes, 4.X.2011 (leaf litter, Afromontane forest), 2♂ (NCA 2012/5502); Kei Mouth, 32°41.280'S, 28°22.484'E, leg. C. Haddad, 6.XII.2005 (leaf litter, coastal dune forest), 1♀ (NCA 2008/1907); Same locality, 32°41.206'S, 28°22.497'E, leg. C. Haddad, 10.VIII.2002 (leaf litter, coastal dune), 1♂ (NCA 2002/414); Lusikisiki district, Mzimhlava River mouth, 31°20'S, 29°40'E, leg. Baddeley, II.1980 (coastal evergreen forest), 1♀ (MRAC 164163). *KwaZulu-Natal Province*: Howick, Shooter's Hill [29°26'S, 30°19'E, 790m a.s.l.], leg. R.F. Lawrence, X.1937, 1♀ (NMSA 2124); Karkloof Nature Reserve, 29°19.1'S, 30°15.5'E, 1325m a.s.l., leg. M. Mostovski, 28.IX–3.X.2005 (yellow pan trap), 2♂ 2♀ (NMSA 21486); Pietermaritzburg, Town Bush [29°36'S, 30°23'E], leg. R.F. Lawrence, IX–XI.1950, 2♂ (NMSA 5513); Same locality, southern slopes of Hogsback, 29°33'S, 30°21'E, 1000m a.s.l., leg. C.E. Griswold & T. Meikle-Griswold, 20.IX.1984 (Berlese extracted leaf litter, native forest), 1♂ (NMSA 24463).

**Figures 72–75.**
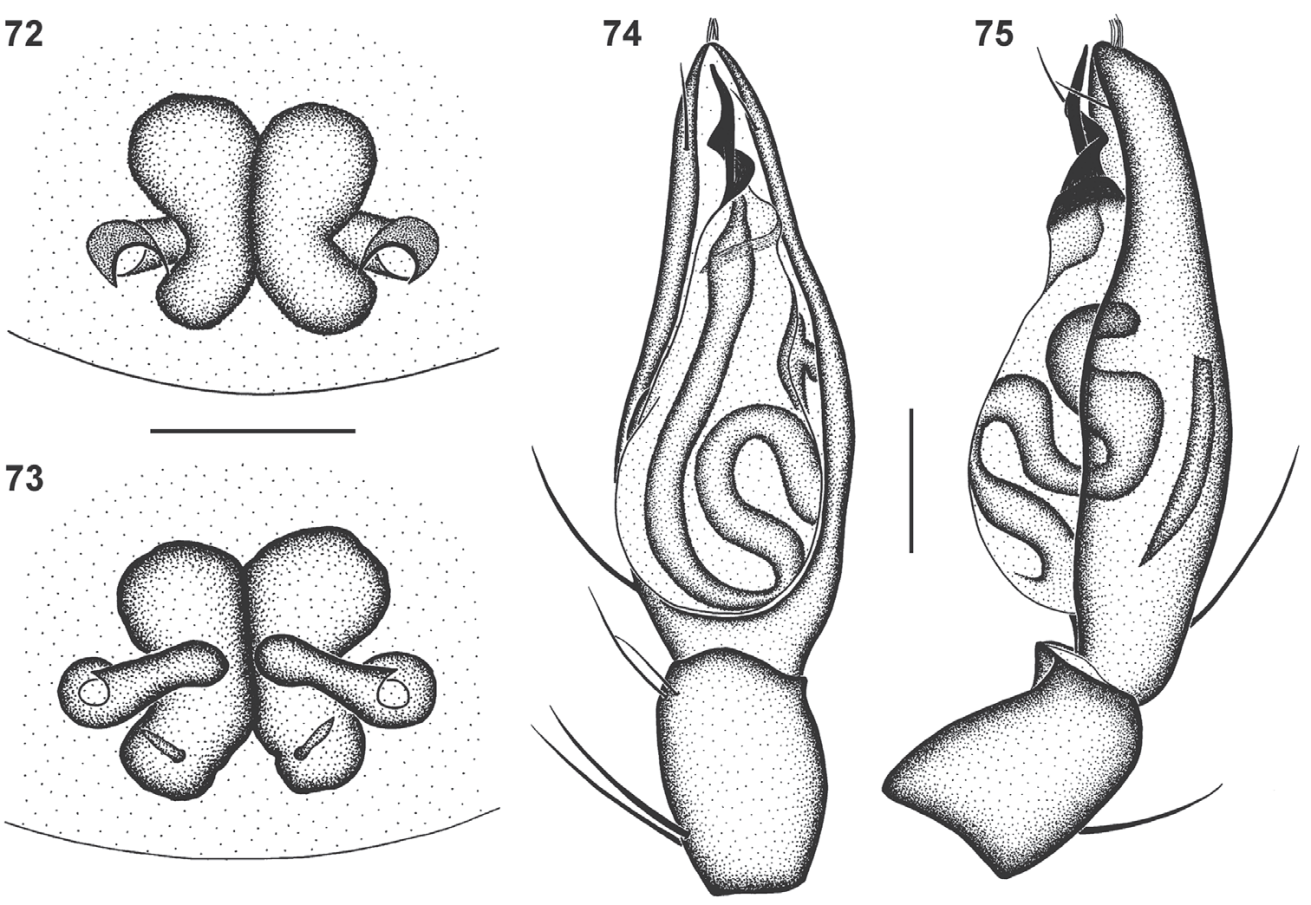
Genitalic morphology of *Copa kei* sp. n.: **72** female epigyne, ventral view **73** same, dorsal view **74** male palp, ventral view **75** same, retrolateral view. Scale bars = 0.25 mm.

**Figure 76.**
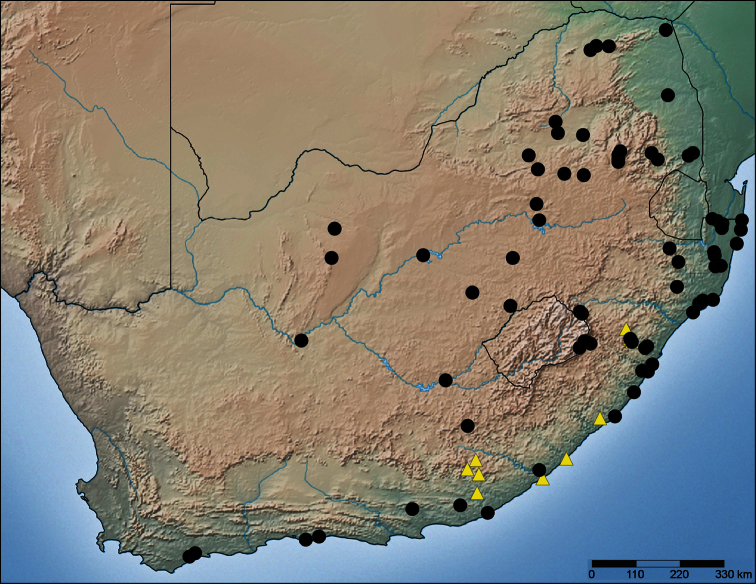
Map of South Africa indicating the distribution of *Copa flavoplumosa* Simon, 1885 (black circles) and *Copa kei* sp. n. (yellow triangles).

**Figures 77–80.**
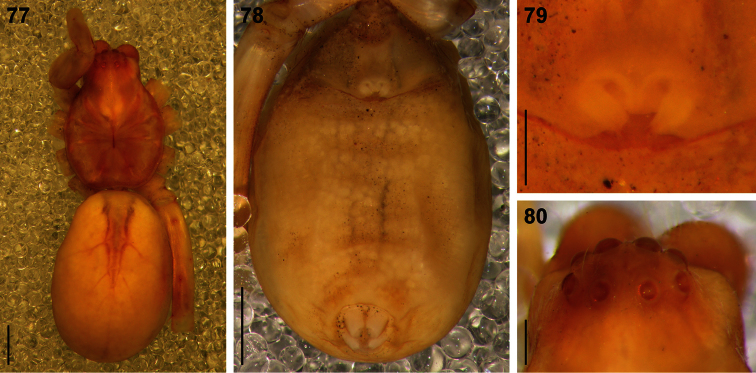
Digital microscope photographs of the holotype subadult female of *Copa agelenina* Simon, 1910: **77** dorsal habitus **78** abdomen, ventral view **79** pre-epigynum **80** eye region, dorsal view. Scale bars: 77, 78 = 1.0 mm; 79, 80 = 0.25 mm.

##### Other material examined.

None.

##### Diagnosis.

The species is easily recognisable by the distinct dorsal black spot on the anterior margin of the abdomen. Males are characterised by the narrow coiled embolus and females by the small copulatory openings and the nearly transverse copulatory ducts.

##### Etymology.

The specific name is a noun in apposition taken from the type locality, the town Kei Mouth, located at the estuary of the Great Kei River in the Eastern Cape Province.

##### Female

**(holotype, Kei Mouth, NCA 2007/3843).** Measurements:CL 3.84, CW 2.75, AL 6.00, AW 4.55, TL 9.65 (6.40–9.80), FL 0.40, SL 1.75, SW 1.60, AME–AME 0.10, AME–ALE 0.01, ALE–ALE 0.46, PME–PME 0.20, PME–PLE 0.13, PLE–PLE 0.63, PERW 0.83, MOQAW 0.44, MOQPW 0.51, MOQL 0.54.

Length of leg segments: I 2.60 + 1.25 + 2.03 + 2.05 + 1.18 = 9.11;II 2.50 + 1.24 + 1.90 + 2.00 + 1.16 = 8.80; III 2.45 + 1.23 + 1.88 + 2.30 + 1.16 = 9.02;IV 3.15 + 1.38 + 2.63 + 3.40 + 1.39 = 11.95.

General appearance as in Fig. 10. Carapace bright yellow-orange, eye region black except between PME; broad median black line covered in black feathery setae from PER to posterior slope of carapace, broken up by asetose line from PME to midpoint and paired oblique asetose line from fovea towards anterior coxae; black striae present, falling within broad median band; lateral margins black from chelicerae to posterior marking, markings expanded from coxae I and from coxae I–IV, densely covered in black feathery setae; areas between markings covered in white feathery setae. All eyes with black rings; AER procurved, medians much larger than laterals; AME separated by distance equal to ½ their diameter; AME separated from ALE by distance approximately ^1^∕_10_ AME diameter; clypeus height approximately 1½ AME diameter; PER strongly procurved, medians very slightly larger than laterals; PME separated by distance equal to 1¼ their diameter; PME separated from PLE by distance equal to ^4^∕_5_ PME diameter; CW:PERW = 3.31:1. Chelicerae yellow-orange, with pectinate curved setae on promargin; three closely spaced teeth on promargin, distal tooth smallest, median tooth largest; median tooth closer to distal tooth than to proximal tooth; retromargin with two teeth separated by their basal width, distal tooth slightly smaller than proximal tooth, close to fang base. Endites yellow, cream prolaterally; labium yellow-brown, cream distally, with broad transverse black marking along proximal margin; sternum bright yellow, with broad black marking along margins, expanded at coxae (Fig. 12). Legs yellow-brown, with faint black mottling; spine bases with distinct black spot; trochanters with distal margins black laterally; femora all with black lateral and distal mottling, ventrally with faint distal ring; patellae with fine dorsal proximal line and lateral and distal mottling; tibiae with faint rings proximally and medially corresponding to ventral spines, distal ends with black ring; metatarsi with proximal, medial and distal rings, corresponding to paired leg spines; tarsi yellow; palp yellow, spines with black spots. Leg spination: femora: I pl 2 do 3 rl 1, II pl 2 do 3 rl 1, III pl 2 do 3 rl 2, IV pl 2 do 3 rl 2; all femora with scattered erect ventral setae; patellae: I & II with fine proximal and distal do setae, III & IV with proximal and distal do spines, proximal spine finer and shorter than distal; tibiae: I do 1 long fine seta, plv 2 rlv 2, II do 1 long fine seta, plv 1 rlv 2 spines, III pl 2 do 1 rl 2 plv 2 rlv 1-2 vt 2, IV pl 2 do 1 rl 2 plv 2 rlv 2 vt 2; metatarsi: I plv 2 rlv 2, II plv 2 rlv 2, III pl 3 rl 3 plv 2 rlv 2 vt 3, IV pl 3 rl 3 plv 2 rlv 2 vt 3. Palpal spination: femora: pl 1 do 2, with scattered erect ventral setae, mainly retrolaterally; patellae: pl 1 do 2; tibiae: pl 1 do 2 plv 1; tarsi: pl 1 rl 1 plv 3 rlv 1. Abdomen with very small red-brown anterior dorsal scutum beneath marking; dorsum mottled grey, with large black spot anteriorly, dark grey median stripe from anterior spot to midpoint, and small cream chevrons posteriorly; short straight black setae and white feathery setae on markings dorsally and laterally; sides of abdomen cream; venter cream, covered in short straight black setae, with dark marking medially on epigastric plate covering epigyne, broadened from epigastric furrow, extending to and surrounding spinnerets. Epigyne small, with strongly curved ridges laterally at midpoint of epigyne, separated by approximately three times their width, with copulatory openings distinct (Figs 57, 72); copulatory ducts almost straight, nearly transverse, slightly oblique, entering rounded anterior ST II; broad ducts connecting ST II to subrectangular posterior ST I; ST I slightly narrower than ST II (Fig. 73).

##### Male

**(paratype, Kei Mouth, NCA 2002/414).** Measurements: CL 3.44, CW 2.54, AL 3.50, AW 2.30, TL 6.98 (5.20–6.98), FL 0.34, SL 1.55, SW 1.48, AME–AME 0.07, AME–ALE 0.01, ALE–ALE 0.40, PME–PME 0.20, PME–PLE 0.11, PLE–PLE 0.55, PERW 0.75, MOQAW 0.42, MOQPW 0.48, MOQL 0.52.

Length of leg segments: I 2.35 + 1.13 + 1.87 + 1.97 + 1.20 = 8.52; II 2.32 + 1.10 + 1.75 + 1.93 + 1.15 = 8.25; III 2.28 + 0.98 + 1.75 + 2.13 + 1.10 = 8.24; IV 2.90 + 1.13 + 2.40 + 3.30 + 1.30 = 11.03.

General appearance as in Fig. 11, male more slender than female. Carapace deep orange, markings and setae as for female. All eyes with black rings; AER procurved, medians much larger than laterals; AME separated by distance equal to ^2^∕_5_ their diameter; AME separated from ALE by distance approximately ^1^∕_10_ AME diameter; clypeus height equal to 1½ AME diameter; PER strongly procurved, medians very slightly larger than laterals; PME separated by distance equal to 1½ their diameter; PME separated from PLE by distance slightly larger than ^4^∕_5_ PME diameter; CW:PERW = 3.39:1. Chelicerae yellow-orange, with curved setae on promargin not pectinate; dentition as for female. Endites, labium, sternum and leg colouration and markings as for female. Leg spination: femora: I pl 2 do 3 rl 1, II pl 2 do 3 rl 1, III pl 2 do 3 rl 2, IV pl 2 do 3 rl 2; all femora with scattered erect ventral setae; patellae: I & II with fine proximal and distal do setae, III & IV with proximal and distal do spines, proximal spine finer and shorter than distal; tibiae: I do 1 long fine seta, plv 3 rlv 3 spines, II do 1 long fine seta plv 2 rlv 3, III pl 2 do 1 rl 2 plv 2 rlv 2 vt 2, IV pl 2 do 1 rl 2 plv 2 rlv 2 vt 2; metatarsi: I plv 2 rlv 2, II plv 2 rlv 2, III pl 3 rl 3 plv 1-2 rlv 1-2 vt 3, IV pl 3 rl 3 plv 2 rlv 2 vt 3. Palpal spination: femora: pl 1 do 2, with scattered erect ventral setae, mainly retrolaterally; patellae: pl 1 do 2; tibiae: pl 1 do 1 plv 1; tarsi: pl 2 rl 1 plv 2 rlv 1. Abdomen with narrow red-brown dorsal scutum extending just past midpoint; dorsum mottled grey, with large black spot anteriorly, broad dark grey median stripe from anterior spot narrowing towards posterior of scutum, and small cream chevrons in posterior half; short straight black setae and white feathery setae on markings dorsally and laterally; lateral margin of abdomen creamy-grey; venter creamy-grey, covered in short straight black setae, with narrow dark grey marking medially on epigastric plate, broadened from epigastric furrow, extending to and surrounding spinnerets. Male palpal cymbium yellow, with several thicker bent setae distally (Fig. 75); tegulum pear-shaped, orange-brown, with nearly black ducts; embolus with narrow base and 1½ narrow coils around a central prong; distal section slightly curved (Figs 58, 66, 74).

##### Distribution.

Known from the south-eastern parts of South Africa (Fig. 76); endemic to the Maputaland-Pondoland-Albany Centre of Endemism (Driver et al. 2005).

##### Biology.

Specimens were mainly collected from the leaf litter layer of closed canopy Afromontane and coastal forest habitats.

### Species *nomen dubium*

#### Copa agelenina

Simon, 1910

Figures 77–80

Copa agelenina Simon 1910: 202.

##### Type material.

**Subadult female holotype.** BOTSWANA: Kalahari, Sekcoma [24°24'S, 23°53'E] – Khakea [24°42'S, 23°30'E], leg. L. Prueltje?, XI.1904, ZMB 28198 (examined).

##### Remarks.

The holotype is a subadult female specimen (Figs 77–79), clearly with a pre-epigynum and lack of epigynal sclerotisation typical of adults, and is not an adult female as described by Simon (1910) and listed on Platnick (2012). The species is definitely different to *Copa flavoplumosa* specimens collected in north-western South Africa (Sunnyside and Hermitage) and has a clearly broader PER (CW:PERW = 2.84:1 as opposed to 3.45:1 in *Copa flavoplumosa* females) that is less strongly procurved than in *Copa flavoplumosa*. Since no adult *Copa* material is available from the arid savanna of southern Botswana, it is impossible to match this specimen to either of the two continental species or to recognise it as a distinct species. I would therefore propose that *Copa agelenina* be considered a species *nomen dubium*.

The eye arrangement and measurements (Fig. 80) suggest that this species may belong to one of the new cryptic lycosiform castianeirine genera (Haddad 2012b, in prep.), but adults will have to be collected before the generic placement can be confirmed and the species be revalidated and redescribed.

## Discussion

The current study treated the continental species of *Copa* in the Afrotropical Region, reducing the number of species from four to two, of which one species is newly described. The type species of the genus, *Copa flavoplumosa*, is widespread throughout the region and includes two synonyms newly proposed here. It is distributed from Guineé in the west to Tanzania in the east, and from Nigeria in the north to South Africa in the south. The new species, *Copa kei*, is endemic to south-eastern South Africa. While *Copa flavoplumosa* provides a useful example of extreme habitat flexibility, occupying habitats from forests to semi-deserts, *Copa kei* is very closely associated with Afromontane and coastal forests in South Africa. The latter species has a distribution falling entirely within the Maputaland-Pondoland-Albany Centre of Endemism in South Africa (Driver et al. 2005). Surprisingly, very few *Copa flavoplumosa* records exist from the tropical rainforests of the D.R. Congo and Congo Republic, despite the former being one of the best sampled countries on the continent (Fig. 71). The two species therefore represent extremes regarding both vagility and ecological flexibility.

Both species are clearly ground-dwelling leaf litter specialists and were mainly collected by pitfall trapping, litter sifting and hand collecting. *Copa flavoplumosa* may be very abundant in some habitats (e.g. forests in Ivory Coast), but they tend to be considerably less common in savannas and other habitat types (Modiba et al. 2005; Dippenaar-Schoeman and Wassenaar 2006; Foord et al. 2008; Haddad et al. 2010; Muelelwa et al. 2010). They have occasionally been collected in agroecosystems, specifically from the canopies of orchard crops in South Africa (avocadoes, macadamias and pistachios), but never exceed 2% of the total spider fauna (Dippenaar-Schoeman et al. 2001, 2005; Haddad et al. 2005). Their arboreal habits in agroecosystems are in stark contrast to their almost exclusive ground-dwelling habits in natural habitats, and the reasons for this ecological divergence are unknown.

## Supplementary Material

XML Treatment for Copa

XML Treatment for Copa flavoplumosa

XML Treatment for Copa kei

XML Treatment for Copa agelenina

## References

[B1] Bosselaers J Jocqué R (2000) Studies in Corinnidae: transfer of four genera and descriptions of the female of *Lessertina mutica* Lawrence 1942. Tropical Zoology 13: 305-325. doi: 10.1080/03946975.2000.10531138

[B2] Deeleman-Reinhold CL (1995) New or little known non-antmimicking spiders of the subfamily Castianeirinae from southeast Asia (Arachnida: Araneae: Clubionidae). Beiträge zur Araneologie 4: 43-54.

[B3] Deeleman-Reinhold CL (2001) Forest spiders of South East Asia: with a revision of the sac and ground spiders (Araneae: Clubionidae, Corinnidae, Liocranidae, Gnaphosidae, Prodidomidae and Trochanterriidae [sic]). Brill, Leiden, 591 pp.

[B4] Dippenaar-Schoeman AS Jocqué R (1997) African spiders: an identification manual.Plant Protection Research Institute Handbook No. 9. Agricultural Research Council, Pretoria, 392 pp.

[B5] Dippenaar-Schoeman AS Van den Berg AM Van den Berg MA Foord SH (2005) Spiders in avocado orchards in the Mpumalanga Lowveld of South Africa: species diversity and abundance (Arachnida: Araneae). African Plant Protection 11: 8-16.

[B6] Dippenaar-Schoeman AS Van den Berg MA Van den Berg AM Van den Berg A (2001) Spiders in macadamia orchards in the Mpumalanga Lowveld of South Africa: species diversity and abundance (Arachnida: Araneae). African Plant Protection 7: 39-46.

[B7] Dippenaar-Schoeman AS Wassenaar TD (2006) A checklist of spiders from the herbaceous layer of a coastal dune forest ecosystem at Richards Bay, KwaZulu-Natal, South Africa (Arachnida: Araneae). African Invertebrates 47: 63-70.

[B8] Driver A Maze K Rouget M Lombard AT Nel J Turpie JK Cowling RM Desmet P Goodman P Harris J Jonas Z Reyers B Sink K Strauss T (2005) National Spatial Biodiversity Assessment 2004: Priorities for Biodiversity Conservation in South Africa. Strelitzia 17. South African National Biodiversity Institute, Pretoria, 44 pp.

[B9] Foord SH Mafadza M Dippenaar-Schoeman AS Van Rensburg BJ (2008) Micro-scale heterogeneity of spiders (Arachnida: Araneae) in the Soutpansberg, South Africa: a comparative survey and inventory in representative habitats. African Zoology 43: 156-174. doi: 10.3377/1562-7020-43.2.156

[B10] Haddad CR (2012a) A revision of the spider genus *Echinax* Deeleman-Reinhold, 2001 (Araneae: Corinnidae) in the Afrotropical Region. Zootaxa 3450: 33-61.

[B11] Haddad CR (2012b) Advances in the systematics and ecology of African Corinnidae spiders (Arachnida: Araneae), with emphasis on the Castianeirinae. Unpublished PhD thesis, Bloemfontein, South Africa: University of the Free State.

[B12] Haddad CR (2012c) A revision of the Afrotropical spider genus *Cambalida* Simon, 1909 (Araneae, Corinnidae). ZooKeys 234: 67-119. doi: 10.3897/zookeys.234.3417 PMC3496914 23372409

[B13] Haddad CR Dippenaar-Schoeman AS Pekár S (2005) Arboreal spiders (Arachnida: Araneae) in pistachio orchards in South Africa. African Plant Protection 11: 32-41.

[B14] Haddad CR Honiball AS Dippenaar-Schoeman AS Slotow R Van Rensburg BJ (2010) Spiders (Arachnida: Araneae) as indicators of elephant-induced habitat changes in the Maputaland Centre of Endemism, South Africa. African Journal of Ecology 48: 446-460. doi: 10.1111/j.1365-2028.2009.01133.x

[B15] Lessert R de (1921) Araignées du Kilimandjaro et du Merou (suite). 4. Clubionidae. Revue Suisse de Zoologie 28: 381-442.

[B16] Lessert R de (1933) Araignées d’Angola. (Resultats de la Mission scientifique suisse en Angola 1928–1929). Revue Suisse de Zoologie 40: 85-159.

[B17] Mansell MW (1996) The antlions of southern Africa (Neuroptera: Myrmeliontidae): genus *Palparellus* Navás, including extralimital species. African Entomology 4: 239-267.

[B18] Modiba MA Dippenaar SM Dippenaar-Schoeman AS (2005) A checklist of spiders from Sovenga Hill, an inselberg in the Savanna Biome, Limpopo Province, South Africa (Arachnida: Araneae). Koedoe 48: 109-115. doi: 10.4102/koedoe.v48i2.95

[B19] Muelelwa MI Foord SH Dippenaar-Schoeman AS Stam EM (2010) Towards a standardized and optimized protocol for rapid assessments: spider species richness and assemblage composition in two savanna vegetation types. African Zoology 45: 273-290. doi: 10.3377/004.045.0206

[B20] Platnick NI (2012) The World Spider Catalog, Version 13.0. American Museum of Natural History, New York. Available from: http://research.amnh.org/entomology-/spiders/catalog [accessed 7 September 2012].

[B21] Reiskind J (1969) The spider subfamily Castianeirinae of North and Central America (Araneae, Clubionidae). Bulletin of the Museum of Comparative Zoology 138: 163-325.

[B22] Shorthouse DP (2010) SimpleMappr, an online tool to produce publication-quality point maps. Available from: http://www.simplemappr.net [accessed 25 May 2012].

[B23] Simon E (1885) Etudes arachnologiques. 18e Mémoire. XXVI. Matériaux pour servir à la faune des Arachnides du Sénégal. (Suivi d’une appendice intitulé: Descriptions de plusieurs espèces africaines nouvelles). Annales de la Société Entomologique de France (6) 5: 345–396.

[B24] Simon E (1896) Descriptions d’arachnides nouveaux de la famille des Clubionidae. Annales de la Société entomologique de Belgique 40: 400-422.

[B25] Simon E (1897) Histoire naturelle des Araignées. Tome second, Premier fascicule. Librairie Encyclopédique de Roret, Paris, 1–192.

[B26] Simon E (1903) Descriptions d’arachnides nouveaux de Madagascar, faisant partie des collections du Muséum. Bulletin du Museum National d’Histoire Naturelle, Paris 9: 133-140.

[B27] Simon E (1909) Arachnides recueillis par L. Fea sur la côte occidentale d’Afrique. 2e partie. Annali del Museo Civico di Storia Naturale di Genova 44: 335-449.

[B28] Simon E (1910) Arachnoidea. Araneae (ii). In: Schultze L (Ed). Zoologische und anthropologische Ergebnisse einer Forschungsreise im Westlichen und zentralen Südafrika. Denkschriften der Medicinish–Naturwissenschaftlichen Gesellschaft zu Jena 16: 175–218.

[B29] Strand E (1907) Diagnosen neuer Spinnen aus Madagaskar und Sansibar. Zoologischer Anzeiger 31: 725-748.

[B30] Strand E (1916) Zentralafrikanische Clubioniden. In: Wissenschaftliche Ergebnisse der Deutschen Zentral Afrika Expedition 1907–1908, unter Fuhrung Adolf Friedrichs, Herzogs zu Mecklenberg. Archiv für Naturgeschichte 81(A11): 79–98.

[B31] Yang JY Song DX Zhu MS (2004) On the newly recorded genus *Echinax* from China (Araneae: Corinnidae), with description of a new species. Journal of the Agricultural University of Hubei 27: 66-70.

